# A unified RT-DETR–Mask2Former framework for segmentation-guided plant leaf disease detection and classification in real-world agricultural environments

**DOI:** 10.3389/fpls.2026.1931819

**Published:** 2026-10-15

**Authors:** C H. Mohan Sai Kumar, Golthu Anusha, V. Dhilip Kumar, Oana Geman, Roxana Toderean, Mihaela Hnatiuc

**Affiliations:** 1 Department of Electronics and Communication Engineering, Vel Tech Rangarajan Dr. Sagunthala R&D Institute of Science and Technology, Chennai, India; 2 Department of General Medicine, Saveetha Institute of Medical and Technical Sciences, Chennai, India; 3 Department of Computer Science and Engineering, Vel Tech Rangarajan Dr. Sagunthala R&D Institute of Science and Technology, Chennai, India; 4 Division of Data Science and Artificial Intelligence, Computer Science and Engineering, Chalmers University of Technology, Gothenburg, Sweden; 5 Division of Data Science and Artificial Intelligence, Computer Science and Engineering, University of Gothenburg, Gothenburg, Sweden; 6 Department of Computers, Electronics and Automation, Stefan cel Mare University of Suceava, Suceava, Romania; 7 Department of Electronic and Telecommunication, Constanta Maritime University, Constanta, Romania

**Keywords:** disease-region isolation, lesion segmentation, Mask2Former, plant leaf disease, RT-DETR, segmentation-guided classification

## Abstract

Plant leaf disease image analysis under real-world field conditions remains challenging due to cluttered backgrounds, illumination variation, overlapping leaves, scale changes, and irregular lesion patterns. This study presents a unified RT-DETR–Mask2Former framework for segmentation-guided plant leaf disease detection and classification. In the proposed pipeline, RT-DETR first localizes leaf regions from complex agricultural images, after which Mask2Former generates fine-grained lesion masks within the detected regions. The refined lesion masks are used to isolate disease-affected regions, enabling EfficientNet-B4 to extract disease-focused feature representations for classification. An EMA-normalized adaptive task-weighting procedure was applied with AdamW optimization to modulate the relative gradient scales of the separately supervised modules. Model development and internal evaluation were conducted using leakage-controlled, group-aware PlantVillage (PV) partitions, whereas 4,200 field-acquired images were reserved exclusively for independent external evaluation. On the complete 38-category PV internal test, accuracy and macro-F1 were 98.34% and 97.97%. On the nine-category source-matched subset, accuracy and macro-F1 were 97.43% and 96.77%, versus 84.93% and 80.80% in the field. PV-to-field performance declined from 95.63% to 89.28% for leaf-localization AP@0.5 and from 92.47%/86.00% to 77.90%/63.80% for lesion-positive Dice/IoU. Lesion guidance improved field macro-F1 from 73.97% to 80.80%. The complete pipeline required approximately 285 GFLOPs and 63 ms per image (16 FPS), demonstrating a practical accuracy–efficiency trade-off while retaining a measurable field-domain gap.

## 1 Introduction

Agriculture remains central to food availability, rural livelihoods, economic development, and long-term sustainability. However, plant diseases continue to reduce crop productivity and quality, creating a strong need for diagnostic systems that are timely, accurate, and scalable. Recent reviews on image-based plant disease recognition indicate that deep learning has become a major direction for automated plant disease diagnosis because of its ability to learn discriminative visual features from leaf images (Abade et al., 2021). Early identification of infected plants is important for timely crop management, targeted pesticide application, and precision agriculture-based decision-making.

Leaves are among the most informative plant organs for visual disease diagnosis because disease symptoms commonly appear as chlorosis, necrosis, discoloration, spots, lesions, mildew structures, and texture irregularities. The study (Mohanty et al., 2016) demonstrated the potential of deep learning for leaf image-based plant disease detection, while the research (Too et al., 2018) showed that fine-tuned deep Convolutional Neural Networks (CNN) architectures can achieve high recognition accuracy on benchmark plant disease datasets. Although such classification models are effective under controlled imaging conditions, they usually operate on complete leaf images and provide limited information about the location, shape, and spatial extent of infected regions.

The increasing use of smart farming technologies, unmanned aerial vehicles, mobile imaging systems, and Internet of Things-enabled agricultural monitoring has further increased the need for automated plant disease analysis methods that can support practical field deployment (Gatkal et al., 2024; Ayaz et al., 2019). The images acquired in the farm are usually very different from those taken under laboratory conditions. They usually contain cluttered backgrounds, shadows, uneven illumination, overlapping foliage, and partially covered leaves. This makes it harder to diagnose diseases, as it requires models that not only correctly classify the diseases but also pinpoint their location on the image.

Deep Learning has made progress in the field of analyzing plant diseases due to automatic learning of features from large sets of images (Barbedo, 2018; LeCun et al., 2015). CNN models like AlexNet, ResNet, and EfficientNet have shown excellent results for visual perception and plant disease classification (Krizhevsky et al., 2017; He et al., 2016; Tan and Le, 2019). However, most classification-based approaches provide only image-level classifications. These approaches do not define boundaries of lesions, which is a drawback when we consider real-world applications like analyzing lesion development and severity.

To improve spatial interpretation, object detection techniques like Faster Region-based Convolutional Neural Networks (R-CNN) and YOLO variations have been used for locating leaves and diseases (Ren et al., 2017; Fuentes et al., 2017; Redmon and Farhadi, 2018; Jocher et al., 2023). Such techniques give bounding-box interpretation and they are better at giving visual interpretability compared to pure image classifiers. However, bounding boxes are still very spatially imprecise and may fail to represent the irregular shape of the lesion, the small infection area or the disease features on overlapping leaves. Techniques that can do pixel-level segmentation like U-Net and Mask R-CNN have been able to solve such challenges (Ronneberger et al., 2015; He et al., 2017). Despite this, in most of the studies done thus far, pixel-level segmentation has been done independently of the final disease classification step.

Recent vision transformers have enhanced the capability to handle long-distance dependencies and global contextual associations even further. Various designs, including Vision Transformers, Swin Transformers, DETR, RT-DETR, and Mask2Former, have shown strong performance in performing tasks like object detection, semantic or instance segmentation and image classification (Liu et al., 2021; Carion et al., 2020; Zhao et al., 2024; Cheng et al., 2022). The RT-DETR algorithm is specifically suitable for the task of real-time leaf localization since it utilizes query-based object detection without anchoring and non-maximum suppression mechanisms (Zhao et al., 2024). The Mask2Former approach, on the other hand, considers segmentation as mask classification while using masked attention to extract object regions (Cheng et al., 2022). Such characteristics are potentially suitable for disease diagnosis tasks where leaf localization and accurate lesion boundaries are required.

The major contributions of this work are summarized as follows:

1. An RT-DETR-based leaf localization stage is configured as a class-agnostic leaf detector that processes each complete image once and generates validation-filtered leaf RoIs for downstream analysis.
2. A Mask2Former–ResNet50 stage processes each retained RoI to generate lesion-probability maps, which are thresholded, refined and projected to the original image coordinates to obtain pixel-level lesion masks.
3. A segmentation-guided representation strategy applies refined predicted masks to normalized EfficientNet-B4 inputs. A complete-RoI fallback handles empty lesion predictions, while channel-attention refinement and multi-RoI probability aggregation support image-level disease classification.
4. A supervision-aware coordinated training procedure activates only losses supported by verified annotations. Exponential-moving-average (EMA) loss normalization and smoothed adaptive task weighting are applied before AdamW updates, while hard RoI selection and mask processing remain nondifferentiable.
5. Leakage-controlled, group-aware PV partitions and a fully held-out field cohort are used for source-separated evaluation. Complete 38-category and source-matched nine-category classification, leaf localization, lesion segmentation, controlled ablations and computational efficiency are assessed independently.

The rest of this paper is structured as follows. Section 2 surveys existing works on plant disease classification, localization, segmentation, transformers-based models for agriculture vision tasks, and multitask learning. In Section 3, the proposed RT-DETR Mask2Former approach is described. Section 4 details the dataset, annotation process, experimental settings, and training setup. Section 5 discusses the quantitative, qualitative, ablation, computational, and statistical analysis. Finally, Section 6 concludes the study and provides possible directions for further research.

## 2 Literature review

Research on automatic disease analysis for plants has evolved from image-level classifiers to models that not only classify but can also localize and explain the presence of diseases. Recently, leaf localization, detection of the diseased area, and lesion segmentation have become important aspects of interpretability in the form of spatial interpretability. Depending upon the methodology used, current state-of-the-art techniques can be divided into five broad categories including CNN-based disease classification, detection-based disease localization, segmentation-based lesion analysis, transformer-based agriculture vision techniques, and multi-task learning-based disease analysis.

### 2.1 CNN-based plant disease classification

Initially, deep learning models for plant-disease diagnosis were based on CNN. According to work (Mohanty et al., 2016), it was demonstrated that using transfer learning on AlexNet and GoogLeNet provided high accuracy in classification using the PV data set. In another study (Too et al., 2018), various CNN architectures were examined such as VGG16, ResNet50, DenseNet, and Inception models where it was shown that deeper models provided better results in diseases recognition.

Further research examined the impact of dataset size, image variability, and transfer learning on classification performance (Barbedo, 2018). The progress of deep learning has significantly supported plant disease detection by allowing CNNs to extract hierarchical visual representations directly from raw images (LeCun et al., 2015). As a result, AlexNet, ResNet, and EfficientNet models have found extensive use as baseline models for plant disease classification due to their capability of detecting disease texture, color variations, edges, and even semantic visual features (Krizhevsky et al., 2017; He et al., 2016; Tan and Le, 2019). Specifically, among the mentioned models, EfficientNet can be considered particularly valuable thanks to its compound scaling approach that considers depth, width, and input resolution (Tan and Le, 2019).

However, CNN-based classifiers generally operate on the whole leaf image and give the output as class labels without showing the position and boundaries of the affected area. Due to this, despite the effectiveness of CNN-based classifiers in disease classification, they have low applicability for monitoring disease progression and assessment of severity.

### 2.2 Detection-based disease localization

Object detection approaches have been proposed in the plant disease identification task because of the poor interpretability of spatial features from classification-only algorithms. The Faster R-CNN, which is a two-stage detector using a region proposal approach, has been widely adopted as an ideal baseline detector (Ren et al., 2017). The research (Fuentes et al., 2017) showed that deep object detectors can identify both disease symptoms and pest-affected regions in leaf images. This type of localization provides stronger visual evidence than classification alone and supports more precise region-level disease analysis.

Single-stage detectors, particularly YOLO detectors, have received many researchers’ attention in agriculture computer vision applications because of their high efficiency and practicality (Redmon and Farhadi, 2018; Jocher et al., 2023). These algorithms performs well in visible disease and leaf detection, especially in simple and moderate imaging environment settings. But these algorithms may not be reliable if there is small disease symptoms, invisible disease symptoms or visually confusing disease symptoms. In addition, the detection results by bounding box are too rough to depict the irregular shape of the disease lesions.

More recently, transformer-based detectors have been explored to overcome some of the limitations of conventional detection architectures. DETR frames object detection as a query-driven set prediction task, eliminating the dependence on manually designed anchor boxes and non-maximum suppression (Carion et al., 2020). However, its slow convergence and relatively high computational cost may limit its use in real-time agricultural applications. RT-DETR improves this aspect by combining an efficient hybrid encoder with a query-based decoder, enabling faster object localization while preserving the advantages of transformer-based detection (Zhao et al., 2024). Even so, detection models mainly provide object-level localization and do not directly use lesion-level segmentation information to support disease classification.

### 2.3 Segmentation-based lesion analysis

Pixel-level segmentation plays an important role in plant disease analysis because it enables precise lesion boundary extraction, improves disease-region interpretability, and supports severity estimation. U-Net has been widely adopted as a baseline segmentation model because its encoder–decoder structure combines high-level semantic information with low-level spatial details through skip connections (Ronneberger et al., 2015). This design helps preserve boundary information, which is essential when lesion regions are small, irregular, or visually similar to healthy tissue.

Mask R-CNN extends the detection capability of Faster R-CNN by adding a mask prediction branch, enabling simultaneous object localization and instance-level segmentation (He et al., 2017). It can be used for detecting disease regions and producing lesion masks. However, multi-stage segmentation models generally incur more computational expenses that might make them less appropriate for real-time agriculture surveillance. On the other hand, recent models like Mask2Former perform segmentation via the mask classification method using masked attention and achieve semantic, instance, and panoptic segmentation (Cheng et al., 2022). This technique is especially applicable for plant disease detection because disease lesions usually have irregular boundaries and texture.

Despite the ability of segmentation models to provide precise lesion masks, most previous research works have not focused on utilizing segmentation outputs in further processing and have considered it only as an end result. Thus, the impact of lesion masks in enhancing feature extraction of disease regions remains insufficiently investigated.

### 2.4 Transformer-based agricultural vision models

Transformer-based models have become important in computer vision because of their ability to model global contextual relationships. Vision Transformer demonstrated that image patches can be processed using self-attention for visual recognition (Dosovitskiy et al., 2021). Swin Transformer improved scalability by introducing hierarchical representation learning and shifted-window attention, making it useful for dense prediction tasks such as detection and segmentation (Liu et al., 2021).

In object detection, DETR introduced a transformer-based set prediction formulation (Carion et al., 2020), while RT-DETR improved real-time performance by reducing the computational overhead associated with transformer-based detection (Zhao et al., 2024). In segmentation, Mask2Former introduced masked attention for universal segmentation and achieved strong mask prediction capability across multiple segmentation settings (Cheng et al., 2022). These architectures provide important foundations for plant disease diagnosis because plant disease analysis requires both global contextual understanding and fine-grained spatial representation.

Recent agricultural studies have begun to explore transformer-based and hybrid CNN–Transformer models for plant disease recognition, crop monitoring, pest detection, and field-level disease analysis. However, most studies still apply transformers to isolated classification or detection tasks. Few works jointly exploit transformer-based localization, lesion segmentation, and segmentation-guided disease classification within a coordinated learning pipeline. RT-DETR and Mask2Former provide complementary capabilities, but their independent use does not fully exploit the relationship among localized leaf regions, lesion masks, and disease-discriminative representation learning.

### 2.5 Multi-task learning for disease analysis

Multi-task learning has been increasingly adopted in computer vision because related tasks can benefit from shared representations. In plant disease analysis, leaf detection, lesion segmentation, and disease classification are closely related. A model that jointly learns these tasks can reduce redundant feature extraction and improve consistency across localization, segmentation, and classification outputs.

However, the direct optimization of the multi-objectives can lead to task imbalance since the losses related to detection, segmentation, and classification typically have different scales and speeds of convergence. This problem makes the fixed weighting of losses result in the dominance of one task in training, thus decreasing the performance of other tasks. Thus, adaptive balancing of losses is critical to multi-task learning. In the described approach, the AdamW with EMA-normalized adaptive task-loss weighting is utilized.

### 2.6 Research gap

From the reviewed literature, several limitations can be identified. First, CNN-based classification methods achieve strong recognition accuracy but lack lesion localization and spatial interpretability. Second, detection-based methods provide bounding-box-level localization but cannot accurately delineate irregular lesion boundaries (Bochkovskiy et al., 2020; Yao et al., 2025). Third, segmentation-based methods generate pixel-level disease masks but are often used independently and are rarely connected to downstream classification. Fourth, transformer-based agricultural models improve contextual feature learning, but most existing studies focus on either classification, detection, or segmentation as isolated tasks (Yao et al., 2025).

Therefore, a unified framework that combines real-time leaf localization, fine-grained lesion segmentation, and segmentation-guided disease classification remains insufficiently investigated. To address this gap, in the proposed framework, RT-DETR first localizes leaf regions from field images, Mask2Former generates fine-grained lesion masks within localized leaf regions, and the resulting masks guide disease-region isolation and feature extraction for final classification. An EMA-normalized adaptive task weighting with AdamW optimization strategy is further employed to balance detection, segmentation, and classification objectives during coordinated multi-module training.

## 3 Methodology

The proposed framework follows a staged localization–segmentation–classification pathway for plant leaf disease analysis. As summarized in **Figure 1**, the deployable inference pathway comprises complete-image preprocessing, class-agnostic RT-DETR leaf localization, predicted region-of-interest (RoI) extraction, Mask2Former lesion segmentation, lesion-mask refinement, lesion-guided feature construction and EfficientNet-B4 classification with channel-attention refinement. The stop-gradient indicators identify confidence filtering, RoI selection, cropping and binary-mask post-processing as non-differentiable routing operations.

**Figure 1:**
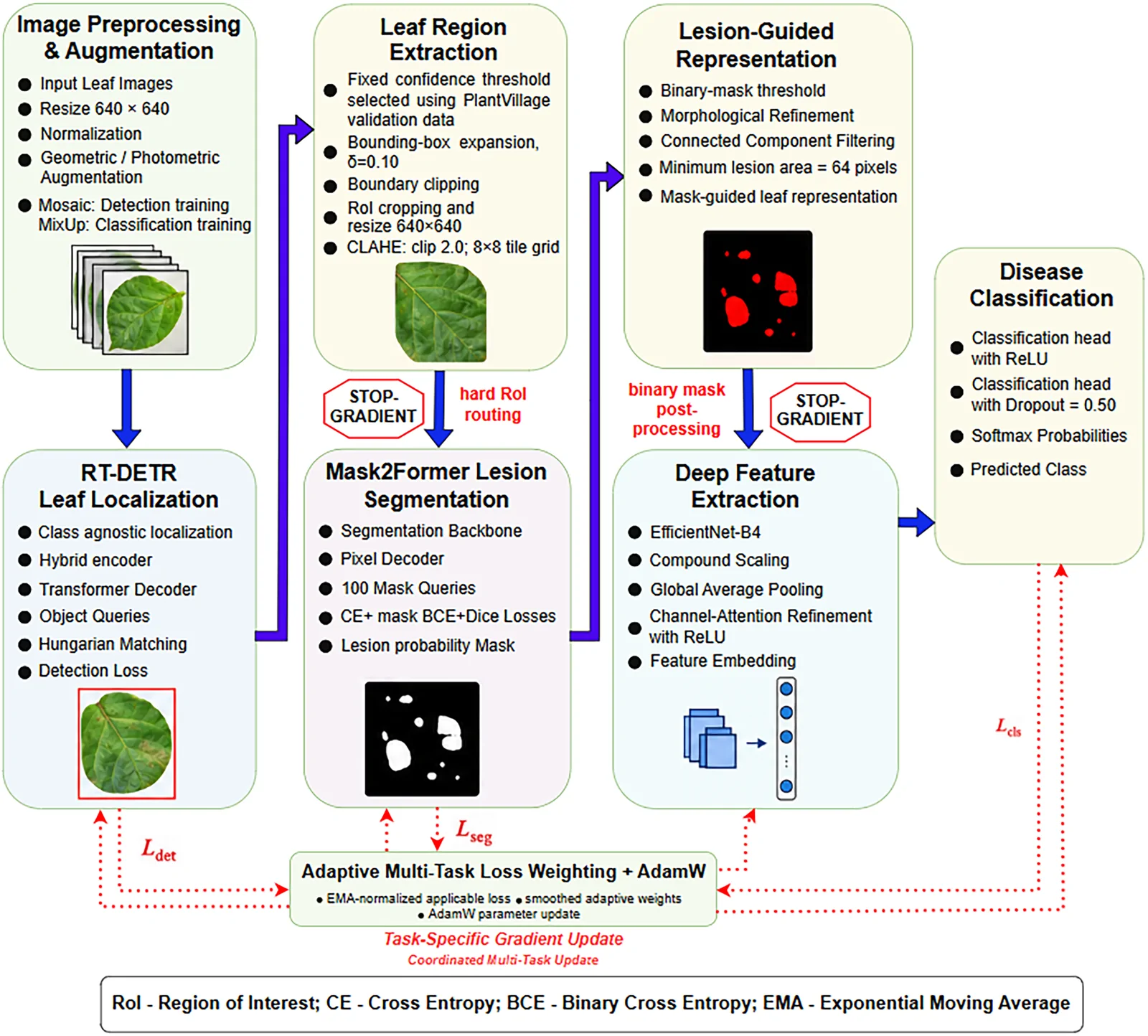
Overall architecture and supervision flow of the proposed RT-DETR–Mask2Former–EfficientNet-B4 framework with channel-attention refinement.

For each complete input image, RT-DETR is executed once at an input resolution of 640×640 pixels and produces *N* = 300 class-agnostic leaf-box predictions with associated confidence scores. Predictions satisfying the detector-confidence threshold and RoI-retention rule selected on the PV validation partition are retained, expanded by the fixed contextual margin and clipped to the image boundaries. Each retained leaf RoI is resized to 640×640 pixels and enhanced using the fixed CLAHE configuration before being processed independently by Mask2Former. The resulting lesion-probability map is thresholded and refined to construct a lesion-guided representation. This representation is subsequently resized to 380×380 pixels before EfficientNet-B4 feature extraction and 38-category disease classification. When an image contains multiple retained RoIs, their probability vectors are combined using the image-level aggregation rule defined in Section 3.7.

Verified leaf bounding boxes, lesion masks and image-level class labels provide supervision only for the corresponding annotated tasks. Samples without bounding-box or mask annotations are excluded from the respective spatial objectives and are not interpreted as negative examples. During validation, internal testing and independent field evaluation, neither ground-truth leaf boxes nor ground-truth lesion masks are supplied to the deployable pathway. Ground-truth RoIs and masks are substituted only in the explicitly identified oracle controls reported in **Table 1**. If no prediction satisfied the frozen detector-confidence and RoI-retention rules, no ground-truth RoI was substituted. For segmentation evaluation, the case was retained as an empty lesion prediction. For end-to-end classification evaluation, the absence of a disease prediction was counted as an incorrect outcome rather than excluding the image from the denominator.

**Table 1:** Oracle and predicted-region control on the fully annotated subsets.

Configuration	Leaf region	Lesion mask	PV macro-F1 (%)	Field macro-F1 (%)
Complete-image control	Complete image	–	89.93	71.03
Oracle-localization control	Ground-truth leaf RoI	–	91.95	76.82
Predicted-localization control	RT-DETR-predicted RoI	–	88.93	73.97
Oracle-mask control		Ground-truth lesion mask	96.50	83.89
Full-pipeline predicted-input evaluation		Predicted Mask2Former mask	94.98	80.80
Full oracle control	Ground-truth leaf RoI	Ground-truth lesion mask	97.99	86.92

Coordinated multi-module training optimizes the applicable task-specific objectives within a common update schedule using disjoint module parameter blocks. The task-activation, stop-gradient and adaptive-weighting procedures are defined formally in Section 3.8; hard RoI selection and binary-mask processing remain non-differentiable.

### 3.1 Image preprocessing and augmentation

Let ${\text{I}}_{i}^{\text{raw}}$ denote a complete three-channel RGB input image of height (*H_i_*) and width (*W_i_*). The original 8-bit image and its intensity-scaled representation are expressed in Equation 1:

(1) $${\text{I}}_{i}^{\left(0\right)}=\frac{{\text{I}}_{i}^{\text{raw}}}{255}\in {\left[0,1\right]}^{H_{i}\times W_{i}\times 3}$$

where ${\text{I}}_{i}^{\left(0\right)}$ denotes the floating-point RGB representation obtained by scaling the original intensity values to [0,1]. Complete images were resized to 640×640 pixels using bilinear interpolation before RT-DETR localization, as specified in **Table 2**. Bounding-box coordinates were transformed analytically under resizing, whereas lesion masks were resized using nearest-neighbor interpolation to preserve discrete mask labels.

**Table 2:** Training, architectural, loss and validation-selected inference configuration of the proposed framework.

Parameter	Value	Parameter	Value
RT-DETR input resolution	640×640	Temperature parameter (τ)	2.0
Mask2Former input resolution	640×640	RT-DETR object queries (*N*)	300
EfficientNet-B4 input resolution	380×380	VFL coefficient, L1​ coefficient, GIoU coefficient $\left(\lambda_{\text{VFL}},\lambda_{L_{1}},\lambda_{\text{GIoU}}\right)$	(1.0, 5.0, 2.0)
Maximum training epochs	150	Mask2Former queries, *K*	100
Training batch size	16	Query-classification coefficient, Binary mask-loss coefficient, Dice-loss coefficient $\left(\lambda_{\text{ce}},\lambda_{\text{bce}},\lambda_{\text{dice}}\right)$	(2.0, 5.0, 5.0)
Base optimizer	AdamW	Bounding-box expansion factor (*δ*)	0.10
Initial learning rate ( $\eta_{\text{max}}$)	1×10^−4^	CLAHE configuration	Clip limit 2.0; 8×8 tile grid
Minimum learning rate ( $\eta_{\text{min}}$)	1×10^−6^	Binary-mask threshold (*τ_m_*)	0.50
Weight decay ( $\lambda_{\text{wd}}$)	1×10^−4^	Minimum lesion area (*A*_min_)	64 pixels
AdamW coefficients $\left(\beta_{1},\beta_{2}\right)$	(0.9, 0.999)	Pooled feature dimension (*C*)	1792
Numerical-stability constant (*ϵ*)	1×10^−8^	Channel-attention reduction ratio (*r_c_*)	16
Learning-rate scheduler	Cosine annealing	Classification-head dropout	0.50
Early-stopping patience	20 epochs	Hidden activation	ReLU
EMA loss-statistic decay (*ρ*)	0.9	Random seed	42
Task-weight smoothing coefficient (*η*)	0.9	Validation frequency	Once per epoch

For an arbitrary RGB tensor $\text{x}\in {\left[0,1\right]}^{H\;\times W\;\times 3}$, the fixed channel-wise normalization operation is defined in Equation 2:

(2) $${\left[N\left(\text{X}\right)\right]}_{c}=\frac{{\text{x}}_{c}-\mu_{c}}{\sigma_{c}+\epsilon },\;c\in \left\{\text{R},\text{G},\text{B}\right\}\text{ and }\epsilon =1\times 10^{-8}.$$

In Equation 2, N(·) denotes channel-wise normalization; X*_c_* is channel *c* of the input tensor; and *μ_c_* and σ*_c_* are the corresponding fixed normalization mean and standard deviation. The term *ϵ* is included as a small constant to ensure numerical stability. The normalization constants were applied unchanged to the training, validation, internal-test and independent field partitions.

Stochastic augmentation was restricted to the PV training partition. Let ${ℛ}_{640}$ denote deterministic resizing to 640×640 pixels, $T_{g}$ denote a sampled geometric transformation and $T_{p}$ denote a sampled photometric transformation. The synchronized transformation of an image and its available spatial annotations is expressed in Equation 3:

(3) $$\begin{array}{c} \left({\text{I}}_{i,640}^{\left(0\right)},\;B_{i,640},{ℳ}_{i,640}\right)={ℛ}_{640}\left({\text{I}}_{i}^{\left(0\right)},\;B_{i},{ℳ}_{i}\right),\\ \left({\text{I}}_{i}^{g},\;B_{i}^{tr},{ℳ}_{i}^{tr}\right)=T_{g}\left({\text{I}}_{i,640}^{\left(0\right)},\;B_{i,640},{ℳ}_{i,640}\right)\text{ and }{\tilde{\text{I}}}_{i}^{tr}=N\left[T_{p}\left({\text{I}}_{i}^{g}\right)\right] \end{array}$$

In Equation 3, $B_{i}$ and ${ℳ}_{i}$ denote the available bounding-box and lesion-mask annotations, respectively; $B_{i,640}$ and ${ℳ}_{i,640}$ are their resized forms; ${\tilde{\text{I}}}_{i}^{tr}$ is the normalized training image; and the superscript tr denotes an augmented training sample. Geometric transformations were applied jointly to images, bounding boxes and masks, whereas photometric transformations modified only RGB intensities. When a spatial annotation was unavailable, it remained unavailable and was not replaced by a negative target.

The geometric augmentation block comprised horizontal flipping with probability 0.50, rotation sampled uniformly from [−15°, +15°], isotropic scaling sampled from [0.80, 1.20] and horizontal and vertical translations of up to 10% of the corresponding image dimension. The photometric block was applied with probability 0.50 and included brightness, contrast and saturation factors sampled from [0.80, 1.20], together with a normalized hue displacement sampled from [−0.05, +0.05]0.

Mosaic augmentation was applied with probability 0.50 only to the RT-DETR detection-training stream. Four training images were combined into a single canvas, and their bounding boxes were remapped, clipped to the mosaic boundaries and checked for non-zero area. Mosaic was not applied to the Mask2Former segmentation stream or the EfficientNet-B4 classification stream.

MixUp was applied with probability 0.20 only to the classification-training stream. For two normalized classification-input tensors ${\text{X}}_{i}^{\text{cls}}$ and ${\text{X}}_{j}^{\text{cls}}$, with corresponding target vectors y*_i_* and y*_j_* the MixUp operation is defined in Equation 4:

(4) $${\text{X}}^{\text{mix}}=\;\text{λ}{\text{X}}_{i}^{\text{cls}}+\left(1-\text{λ}\right){\text{X}}_{j}^{\text{cls}}\;{\text{y}}^{\text{mix}}=\;\text{λ}{\text{y}}_{i}+\left(1-\text{λ}\right){\text{y}}_{j\;}\;\text{λ}\;\sim \;\text{B}et\text{a}\left(0.2,0.2\right)$$

In Equation 4, λ∈[0,1] is the sampled mixing coefficient; **X**^mix^ is the resulting mixed classification input; and **y**^mix^ is the corresponding soft 38-category target satisfying $\Sigma_{c=1}^{38}{\text{y}}_{c}^{\text{mix}}=1$. MixUp was not applied to bounding-box or lesion-mask targets. PV validation, PV internal-test and independent field images underwent deterministic resizing and normalization without stochastic augmentation. CLAHE was not applied to the complete input image. Instead, it was applied in RGB intensity space only after leaf-RoI extraction, as described in Section 3.3.

As summarized in **Table 2**, each retained leaf RoI was resized to 640×640 pixels before Mask2Former segmentation. Following mask refinement and lesion-guided region construction, the corresponding classifier input was resized to 380×380 pixels before EfficientNet-B4 feature extraction, as described in Section 3.6. The complete task-specific preprocessing and augmentation pathway is illustrated in **Figure 2**.

**Figure 2:**
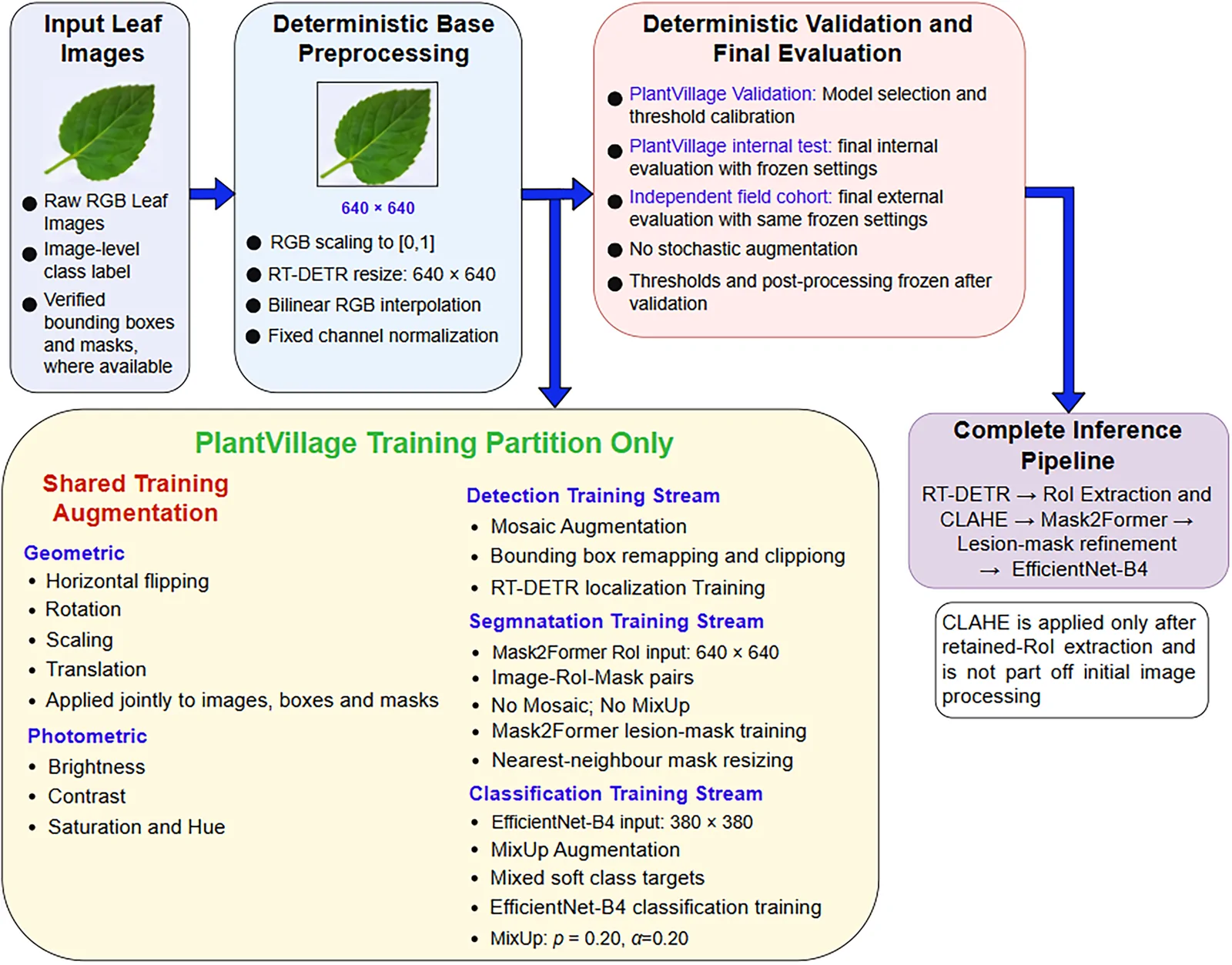
Task-specific image preprocessing and augmentation workflow.

### 3.2 RT-DETR-based leaf localization

The detector receives the normalized full-image tensor ${\text{I}}_{i}^{\text{det}}\in {\mathbb{R}}^{3\times 640\times 640}$ produced by the preprocessing procedure defined in Equations 1–3, where *i* indexes an input image. Leaf localization is formulated as a single-foreground-class detection problem; therefore, the detector distinguishes leaf objects from the no-object condition without predicting disease categories. The complete localization architecture and its training-only supervision paths are shown in **Figure 3**.

**Figure 3:**
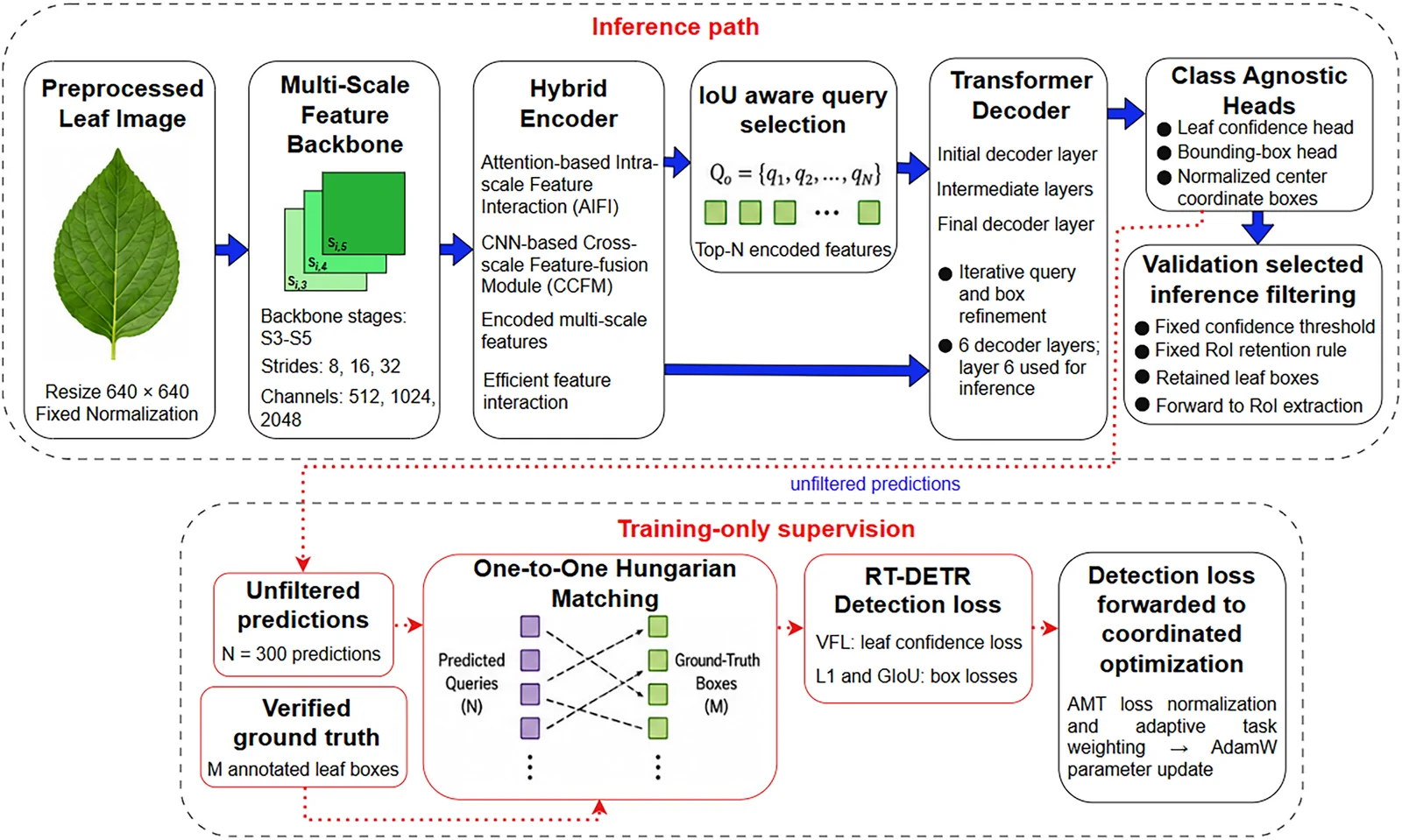
RT-DETR-based class-agnostic leaf-localization architecture.

The localization module used the RT-DETR configuration with the last three backbone stages producing multiscale feature maps as defined in Equation 5:

(5) $${\left\{{\text{S}}_{i,r}\right\}}_{r=3}^{5}=B_{\theta_{b}}\left({\text{I}}_{i}^{\text{det}}\right),\;{\text{S}}_{i,r}\in {\mathbb{R}}^{C_{l}\times H_{l}\times W_{l}},\;r\in \left\{3,4,5\right\}$$

where $B_{\theta_{b}}$ denotes the backbone with parameters *θ_b_*. The feature-level strides are $s_{r}\in \left\{8,16,32\right\}$, giving spatial dimensions (*H_r_*, *W_r_*) = (80,80), (40,40) and (20,20), respectively, for the 640×640-pixel detector input. The corresponding backbone channel dimensions are $C_{r}\in \left\{512,\;1024,\;2048\right\}$.

As illustrated in **Figure 3**, the efficient hybrid encoder separates attention-based intra-scale interaction from convolution-based cross-scale fusion. The highest-level feature map *S_i_*_,5_ is projected to the common hidden dimension *d* = 256, flattened and combined with its two-dimensional positional encoding. The resulting sequence is processed by one Attention-based Intra-scale Feature Interaction (AIFI) layer. The attention-enhanced representation is subsequently fused with the lower-level backbone features through the CNN-based Cross-scale Feature-fusion Module (CCFM), as expressed by Equation 6:

(6) $$\begin{array}{c} {\text{X}}_{i,5}=\text{Flatten}\left({\text{P}}_{5}\left({\text{S}}_{i,5}\right)\right)+{\text{P}}_{i,5},\;{\text{U}}_{i,5}=\text{L}\text{N}\left[{\text{X}}_{i,5}+\text{M}\text{H}\text{A}\left({\text{X}}_{i,5}\right)\right],\\ {\text{F}}_{i,5}=\text{Reshape}\left\{\text{L}\text{N}\left[{\text{U}}_{i,5}+\text{F}\text{F}\text{N}\left({\text{U}}_{i,5}\right)\right]\right\},\;\left\{{\text{Z}}_{i,3},{\text{Z}}_{i,4},{\text{Z}}_{i,5}\right\}=\text{C}\text{C}\text{F}\text{M}\left({\text{P}}_{3}\left({\text{S}}_{i,3}\right),{\text{P}}_{4}\left({\text{S}}_{i,4}\right),{\text{F}}_{i,5}\right) \end{array}$$

Here, $P_{r}\left(\cdot \right)$ denotes the projection of feature level *r* to *d* = 256 channels; P*_i_*_,5_ is its positional encoding; LN(·) denotes layer normalization; MHA(·) is multi-head self-attention; and FFN(·) is the feed-forward network. The encoded feature maps Z*_i_*_,3_, Z*_i_*_,4_ and Z*_i_*_,5_ retain strides of 8, 16 and 32, respectively, and form the multiscale memory supplied to the decoder.

For attention head $h\in \left\{1,\ldots .,H_{a}\right\}$ the scaled dot-product attention and multi-head aggregation are defined in Equation 7:

(7) $$\begin{array}{c} \text{Att}{\text{n}}_{h}\left({\text{X}}_{i,5}\right)=\text{Softmax}\left[\frac{\left({\text{X}}_{i,5}{\text{W}}_{h}^{Q}\right){\left({\text{X}}_{i,5}{\text{W}}_{h}^{K}\right)}^{\text{T}}}{\sqrt{d_{head}}}\right]\left({\text{X}}_{i,5}{\text{W}}_{h}^{V}\right),\\ \text{MHA}\left({\text{X}}_{i,5}\right)=\text{Concat}\left(\text{Att}{\text{n}}_{1}\left({\text{X}}_{i,5}\right),\ldots .Attn_{H_{a}}\left({\text{X}}_{i,5}\right)\right){\text{W}}^{O} \end{array}$$

In Equation 7, *H_a_* = 8 is the number of attention heads; $d_{head}=d/H_{a}=32$ is the key dimension per head; and ${\text{W}}_{h}^{Q}$, ${\text{W}}_{h}^{K}$, ${\text{W}}_{h}^{V}$ and W° are trainable query, key, value and output projection matrices, respectively. The AIFI feed-forward dimension was 1024, with GELU activation and zero attention dropout.

The encoded maps are flattened and concatenated to form $\;{\text{z}}_{i}^{\text{enc}}=\text{Concat}\left[\text{Flatten}\left({\text{Z}}_{i,3}\right),\text{Flatten}\left({\text{Z}}_{i,4}\right),\text{Flatten}\left({\text{Z}}_{i,5}\right)\right]\in {\mathbb{R}}^{T\times d}$, where $T=80^{2}+40^{2}+20^{2}=8400$ is the total number of encoded spatial locations. An encoder prediction head generates a leaf-confidence score $p_{i,t}^{enc}$ and a normalized bounding-box estimate ${\hat{b}}_{i,t}^{enc}$ for every encoded location t∈{1,….,T}. The N = 300 highest-scoring encoder locations initialize the decoder queries and reference boxes, as expressed by Equation 8:

(8) $$\begin{array}{c} {ℐ}_{i,N}^{\text{enc}}=\text{TopNIndices}\left({\left\{p_{i,t}^{\text{enc}}\right\}}_{t=1}^{T}\right),\;{\text{Q}}_{i}^{\left(0\right)}={\text{Z}}_{i}^{\text{enc}}\left[{ℐ}_{i,N}\right]\\ {\hat{B}}_{i}^{\left(0\right)}={\hat{B}}_{i}^{\text{enc}}\left[{ℐ}_{i,N}\right],\;N=300 \end{array}$$

Here, ${ℐ}_{i,N}^{\text{enc}}$ is the ordered index set of the *N* selected encoder locations; ${\text{Q}}_{i}^{\left(0\right)}\in {\mathbb{R}}^{N\times d}$ contains the initial decoder content queries; and ${\hat{B}}_{i}^{\left(0\right)}\in {\left[0,1\right]}^{N\times 4}$ contains their initial reference boxes. Varifocal supervision aligns encoder confidence with localization quality, thereby favoring features that provide both high leaf confidence and reliable spatial localization.

The transformer decoder contains *D* = 6 layers. Each layer refines the query representations, confidence scores and reference boxes using the encoded multiscale memory, as defined in Equation 9:

(9) $$\begin{array}{c} \left({\text{Q}}_{i}^{\left(l\right)},{\hat{p}}_{i}^{\left(l\right)},{\hat{B}}_{i}^{\left(l\right)}\right)=D_{l}^{\text{det}}\left({\text{Q}}_{i}^{\left(l-1\right)},{\hat{\text{B}}}_{i}^{\left(l-1\right)},{\text{Z}}_{i}^{enc}\right),\;l=1,\;\ldots ,\;D_{\text{det}},\\ {\hat{y}}_{i}^{\text{det}}={\left\{\left({\hat{p}}_{i,n}^{\left(D_{\text{det}}\right)},{\hat{b}}_{i,n}^{\left(D_{\text{det}}\right)}\right)\right\}}_{n=1}^{N} \end{array}$$

In Equation 9, $D_{l}^{\text{det}}$ denotes decoder layer *l*; ${\text{Q}}_{i}^{\left(l\right)}$ is its refined query representation; ${\hat{p}}_{i}^{\left(l\right)}\in {\left[0,1\right]}^{N}$ contains sigmoid leaf-confidence scores; and ${\hat{B}}_{i}^{\left(l\right)}\in {\left[0,1\right]}^{N\times 4}$ contains the corresponding boxes. The prediction set ${\hat{y}}_{i}^{\text{det}}$ is obtained from the sixth decoder layer. Each predicted or ground-truth box uses the normalized center-coordinate representation defined in Equation 10:

(10) $${\hat{\text{b}}}_{i,n}^{\left(D_{\text{det}}\right)}=\left({\hat{c}}_{x,i,n}^{\left(D_{\text{det}}\right)},{\hat{c}}_{y,i,n}^{\left(D_{\text{det}}\right)},{\hat{w}}_{i,n}^{\left(D_{\text{det}}\right)},{\hat{h}}_{i,n}^{\left(D_{\text{det}}\right)}\right)\in {\left[0,1\right]}^{N\times 4}$$

where ${\hat{c}}_{x}$ and ${\hat{c}}_{y}$ are the box-center coordinates normalized by image width and height, and $\hat{w}$ and $\hat{h}$ are the normalized box width and height. The same representation is used for ${\hat{b}}_{i,n}^{\left(l\right)},{\hat{b}}_{i,t}^{\text{enc}}$ and the ground-truth box b*_i_*,*_j_*, thereby maintaining consistent notation throughout query selection, decoding, matching and loss calculation.

Let ${\left\{\left(y_{i,j}^{\text{det}},{\text{ b}}_{i,j}\right)\right\}}_{j=1}^{M_{i}}$ denote the verified detection annotations for image *i*, where *M_i_* is the number of ground-truth leaf instances, $y_{i,j}^{\text{det}}=1$ is the class-agnostic leaf target and b*_i_*,*_j_* is its normalized box. For a standard supervised output branch *χ*, one-to-one assignment is obtained by minimizing the Hungarian matching cost defined in Equation 11:

(11) $$\omega_{\text{cls}}C_{\text{cls}}\left(y_{i,j},{\hat{p}}_{i,\pi \left(j\right)}^{\left(\chi \right)}\right)+\omega_{L1}{||{\hat{b}}_{i,\pi \left(j\right)}^{\left(\chi \right)}-b_{i,j}||}_{1}+\omega_{\text{GloU}}\left(1-\text{GloU}\left({\hat{b}}_{i,\pi \left(j\right)}^{\left(\chi \right)},b_{i,j}\right)\right)$$

where $\pi_{N,M_{i}}$ denotes the set of injective assignments from the *M_i_* ground-truth instances to the *N* predictions; *π*(*j*) is the prediction assigned to ground-truth instance *j*; and *C*_cls_ is the focal-style classification matching cost. The matching coefficients were $\omega_{\text{cls}}=2$, $\omega_{\text{L}1}=5$ and $\omega_{\text{GIoU}}=2$, with matcher parameters $\alpha_{\text{match}}=0.25$ and $\gamma_{\text{match}}=2.0$. Hungarian assignment was independently calculated for the final decoder output, each of the five intermediate decoder outputs and the encoder proposal output. Denoising queries used their known constructed target correspondences and therefore did not require Hungarian matching.

For every supervised branch *χ*, the branch-specific detection loss and complete localization objective are defined in Equation 12:

(12) $${ℒ}_{\text{det}}=\sum_{\chi \in {ℛ}_{det}}\gamma_{r}\left[{\text{λ}}_{VFL}{ℒ}_{\text{VFL}}^{\left(\chi \right)}+{\text{λ}}_{\text{L}1}{ℒ}_{\text{L}1}^{\left(\chi \right)}+{\text{λ}}_{\text{GloU}}{ℒ}_{\text{GloU}}^{\left(\chi \right)}\right]$$

In Equation 12, ${ℒ}_{VFL}$ is the Varifocal Loss, ${ℒ}_{L1}$ is the *L*1 bounding-box regression loss and ${ℒ}_{GIoU}$ is the generalized-IoU loss. Varifocal Loss used $\alpha_{VFL}=0.75$ and $\gamma_{VFL}=2.0$. The final sixth-layer decoder loss, the five intermediate decoder auxiliary losses, the encoder proposal loss and the six decoder-layer denoising losses were all enabled. Each branch used the same component coefficients and a branch weight of 1.0; therefore, the branch weights must not be interpreted as replacements for the component coefficients (1.0, 5.0, 2.0).

Contrastive denoising was used only during training with a denoising-query budget of 100, label-noise ratio of 0.5 and box-noise scale of 1.0. Detection loss was computed only for samples containing verified bounding-box annotations; missing detection annotations were excluded rather than treated as background-only targets.

During inference, denoising and all auxiliary loss branches were disabled. Only the sixth decoder-layer predictions were retained, and RT-DETR produced the detection set without non-maximum suppression. The fixed detector-confidence threshold and RoI-retention rule specified in Section 4.6 were then applied before the coordinate-conversion and RoI-extraction procedure described in Section 3.3.

### 3.3 Leaf-region retention and RoI preparation

Only predictions from the final RT-DETR decoder layer *D* = 6 were considered for downstream processing. The detector-confidence threshold $\tau_{\text{det}}=0.50$ and maximum retained-RoI count $R_{\max}=300$, calibrated under the validation-only procedure in Section 4.6, were applied to the final decoder-layer predictions. For image *i*, the retained query-index set is defined in Equation 13:

(13) $$\begin{array}{c} {ℐ}_{i}^{\text{roi}}=\text{To}{\text{p}}_{R_{\text{max}}}\left(\left\{n\in \left\{1,\;\ldots ,\;N\right\}|\;{\hat{p}}_{i,n}^{\left(D_{\text{det}}\right)}\geq \tau_{\text{det}}\right\};{\hat{p}}_{i,n}^{\left(D_{\text{det}}\right)}\right),\\ N_{i}^{\text{roi}}=\;\left|{ℐ}_{i}^{\text{roi}}\right|,\;N=300,\text{ D}=6 \end{array}$$

In Equation 13, ${\hat{p}}_{i,n}^{D}$ is the class-agnostic leaf-confidence score produced by query *n* at the final decoder layer, as defined in Equation 9. The operator ${\text{Top}}_{R_{\text{max}}}\left(\cdot \right)$ sorts threshold-qualified detections in descending confidence order and retains at most *R*_max_ predictions. The set ${ℐ}_{i}^{\text{roi}}$ contains the retained query indices, and $N_{i}^{\text{roi}}$ is the number of RoIs processed for image *i*. Because *R*_max_ = *N* = 300, every threshold-qualified final-layer prediction was retained.

For retained query $n\in {ℐ}_{i}^{\text{roi}}$, let $\left({\hat{c}}_{x,i,n},\;{\hat{c}}_{y,i,n},{\hat{w}}_{i,n},{\hat{h}}_{i,n}\right)$ denote the normalized center-format box defined in Equation 10. For an original image having width *W_i_* and height *H_i_*, the box is converted to continuous pixel-coordinate corner form according to Equation 14:

(14) $$\begin{array}{c} x_{i,n}^{-}=W_{i}\left({\hat{c}}_{x,i,n}^{\left(D_{\text{det}}\right)}-\frac{{\hat{w}}_{i,n}^{\left(D_{\text{det}}\right)}}{2}\right),\;x_{i,n}^{+}=W_{i}\left({\hat{c}}_{x,i,n}^{\left(D_{\text{det}}\right)}+\frac{{\hat{w}}_{i,n}^{\left(D_{\text{det}}\right)}}{2}\right),\;\\ y_{i,n}^{-}=H_{i}\left({\hat{c}}_{y,i,n}^{\left(D_{\text{det}}\right)}-\frac{{\hat{h}}_{i,n}^{\left(D_{\text{det}}\right)}}{2}\right),\;y_{i,n}^{+}=H_{i}\left({\hat{c}}_{y,i,n}^{\left(D_{\text{det}}\right)}+\frac{{\hat{h}}_{i,n}^{\left(D_{\text{det}}\right)}}{2}\right),\;\\ w_{i,n}^{\text{px}}=x_{i,n}^{+}-x_{i,n}^{-}\text{ and }h_{i,n}^{\text{px}}=y_{i,n}^{+}-y_{i,n}^{-} \end{array}$$

Here, $x_{i,n}^{-}$, $y_{i,n}^{-}$, $x_{i,n}^{+}$ and $y_{i,n}^{+}$ are the left, upper, right and lower box boundaries, respectively. The variables $w_{i,n}^{\text{px}}$ and $w_{i,n}^{\text{px}}$ denote the box width and height in original-image pixels. Equation 14 applies to the direct-resize detector preprocessing defined in Equation 3, for which no aspect-ratio padding was introduced.

To preserve limited contextual information around the detected leaf, every retained box was expanded by δ=0.10 of its width and height on each side. Integer crop boundaries were obtained using outward rounding and clipping, as expressed by Equation 15:

(15) $$\begin{array}{c} {\underline{x}}_{i,n}=\;\text{max}\left[0,\left[x_{i,n}^{-}-\delta w_{i,n}^{\text{px}}\right]\right],\;{\underline{y}}_{i,n}=\;\text{max}\left[0,\left[y_{i,n}^{-}-\delta h_{i,n}^{\text{px}}\right]\right]\\ {\bar{x}}_{i,n}=\;\text{min}\left[W_{i},\left[x_{i,n}^{+}+\delta w_{i,n}^{\text{px}}\right]\right],\;{\bar{y}}_{i,n}=\;\text{min}\left[H_{i},\left[y_{i,n}^{+}+\delta h_{i,n}^{\text{px}}\right]\right]\\ {\text{b}}_{i,n}^{\delta }=\left({\underline{x}}_{i,n},{\underline{y}}_{i,n},{\bar{x}}_{i,n},\;{\bar{y}}_{i,n}\right) \end{array}$$

In Equation 15, ⌊·⌋ and ⌈·⌉ denote floor and ceiling operations. The crop follows the half-open array-indexing convention $\left[{\underline{x}}_{i,n},\;{\bar{x}}_{i,n}\right)\times \left[{\underline{y}}_{i,n},\;{\bar{y}}_{i,n}\;\right)$ which prevents ambiguity at the right and lower image boundaries. Because the margin is added independently on both sides, an unclipped expanded box is 20% wider and 20% higher than the corresponding detected box.

CLAHE requires intensity-domain data and therefore was not applied to the normalized detector tensor ${\text{I}}_{i}^{\text{det}}$. Instead, the expanded region was cropped from the original 8-bit RGB image ${\text{I}}_{i}^{\text{raw}}$ and resized to the 640×640-pixel Mask2Former reference grid using bilinear interpolation, as defined in Equation 16:

(16) $$\begin{array}{c} {\text{C}}_{i,n}^{640}=\text{Resiz}{\text{e}}_{\text{bilinear}}\left[\text{Crop}\left({\text{I}}_{i}^{\text{raw}},{\text{b}}_{i,n}^{\delta }\right);\left(640,640\right)\right]\\ {\text{Φ}}_{i,n}\left(u,v\right)=\left({\underline{x}}_{i,n}+\frac{u}{640}\left({\bar{x}}_{i,n}-{\underline{x}}_{i,n}\right),{\underline{y}}_{i,n}+\frac{v}{640}\left({\bar{y}}_{i,n}-y_{i,n}\right)\right) \end{array}$$

Here, ${\text{C}}_{i,n}^{640}$ is the resized 8-bit RoI, and (*u*, *v*) denotes a location on its 640×640-pixel grid. The ${\text{Φ}}_{i,n}$ records the transformation from the Mask2Former RoI grid to the original image coordinates and is retained for subsequent projection of the predicted lesion mask.

Contrast-limited adaptive histogram equalization was applied to the luminance channel of ${\text{C}}_{i,n}^{640}$, using a clip limit of 2.0 and an 8×8 tile grid. The enhanced RGB RoI was then unit-scaled, resized when required and normalized using the operator N(·) defined in Equation 2. The two branch-specific tensors are defined in Equation 17:

(17) $$\begin{array}{c} {\text{E}}_{i,n}^{640}=\text{CLAH}{\text{E}}_{L^{*}}\left({\text{C}}_{i,n}^{640};\text{clip}=2.0,\text{grid}=8\times 8\right),\\ {\text{R}}_{i,n}^{\text{seg}}=N\left(\frac{{\text{E}}_{i,n}^{640}}{255}\right)\in {\mathbb{R}}^{3\times 640\times 640},\\ {\text{R}}_{i,n}^{\text{cls}}=N\left[\frac{\text{Resiz}{\text{e}}_{\text{bilinear}}\left({\text{E}}_{i,n}^{640};\left(380,380\right)\right)}{255}\right]\in {\mathbb{R}}^{3\times 380\times 380},\;n\in {ℐ}_{i}^{\text{roi}} \end{array}$$

In Equation 17, ${\text{CLAHE}}_{L^{*}}\left(\cdot \right)$ denotes conversion from RGB to CIELAB, application of CLAHE to the L^*^ luminance channel and conversion back to RGB. The tensor ${\text{R}}_{i,n}^{\text{seg}}$ is supplied to Mask2Former, whereas ${\text{R}}_{i,n}^{\text{cls}}$ is supplied to EfficientNet-B4. Thus, Mask2Former receives 640×640-pixel RoIs, while EfficientNet-B4 receives its standard 380×380-pixel input. Both tensors originate from the same expanded and CLAHE-enhanced leaf region.

Each retained RoI was processed independently using shared downstream model weights. When $N_{i}^{\text{roi}}=0$, no ground-truth box was substituted and neither downstream module was invoked. The corresponding case remained in the end-to-end evaluation as a localization failure rather than being excluded. Ground-truth RoIs were used only for the oracle controls reported in **Table 1**.

### 3.4 Mask2Former-based lesion segmentation

For input image *i*, the *n^th^* leaf RoI retained by RT-DETR, where $n\in {\text{I}}_{i}^{\text{roi}}$, was represented by the normalized segmentation tensor ${\text{R}}_{i,n}^{\text{seg}}\in {\mathbb{R}}^{3\times 640\times 640}$, as defined in Equation 17 and illustrated in **Figure 4**. Each retained RoI was processed independently using the same Mask2Former parameters. Consequently, processing multiple RoIs increased the number of model invocations but did not duplicate the segmentation parameters.

**Figure 4:**
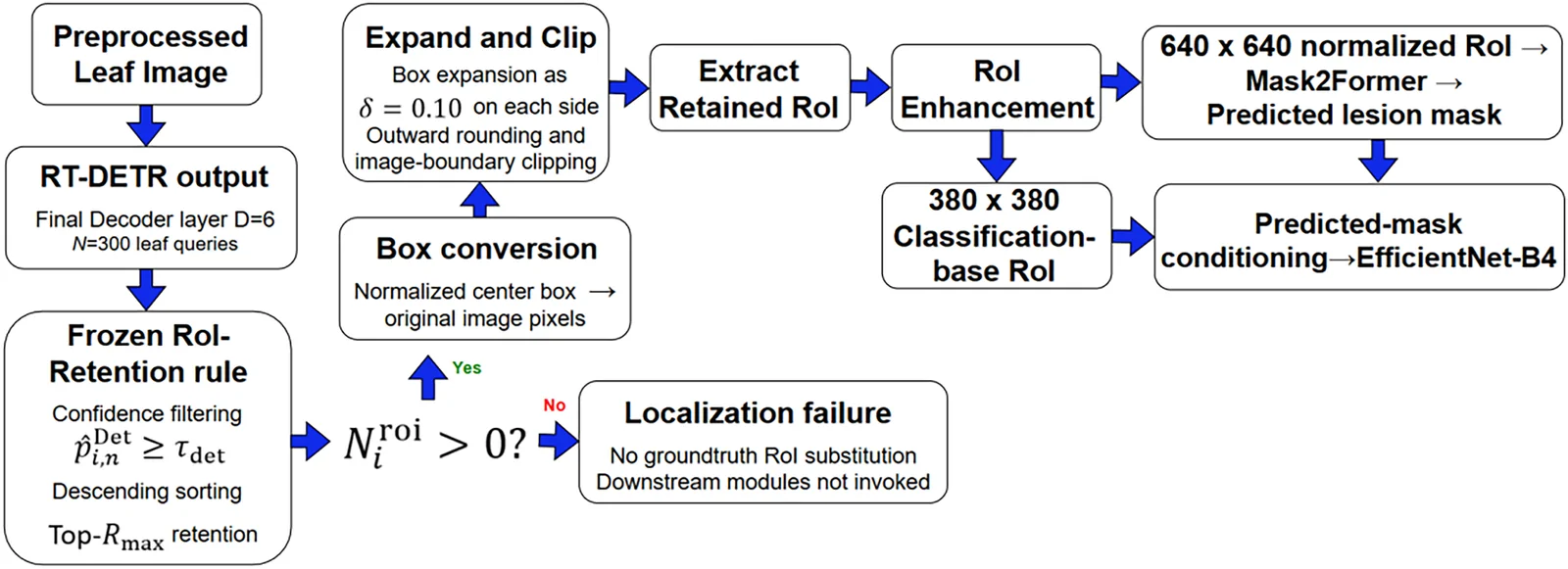
Leaf-region retention, extraction and branch-specific RoI preparation.

The Mask2Former architecture comprised a hierarchical image backbone, a multiscale pixel decoder and a six-layer masked-attention transformer decoder with *K* = 100 segmentation queries. The corresponding training and inference pathways are illustrated in **Figure 5**.

**Figure 5:**
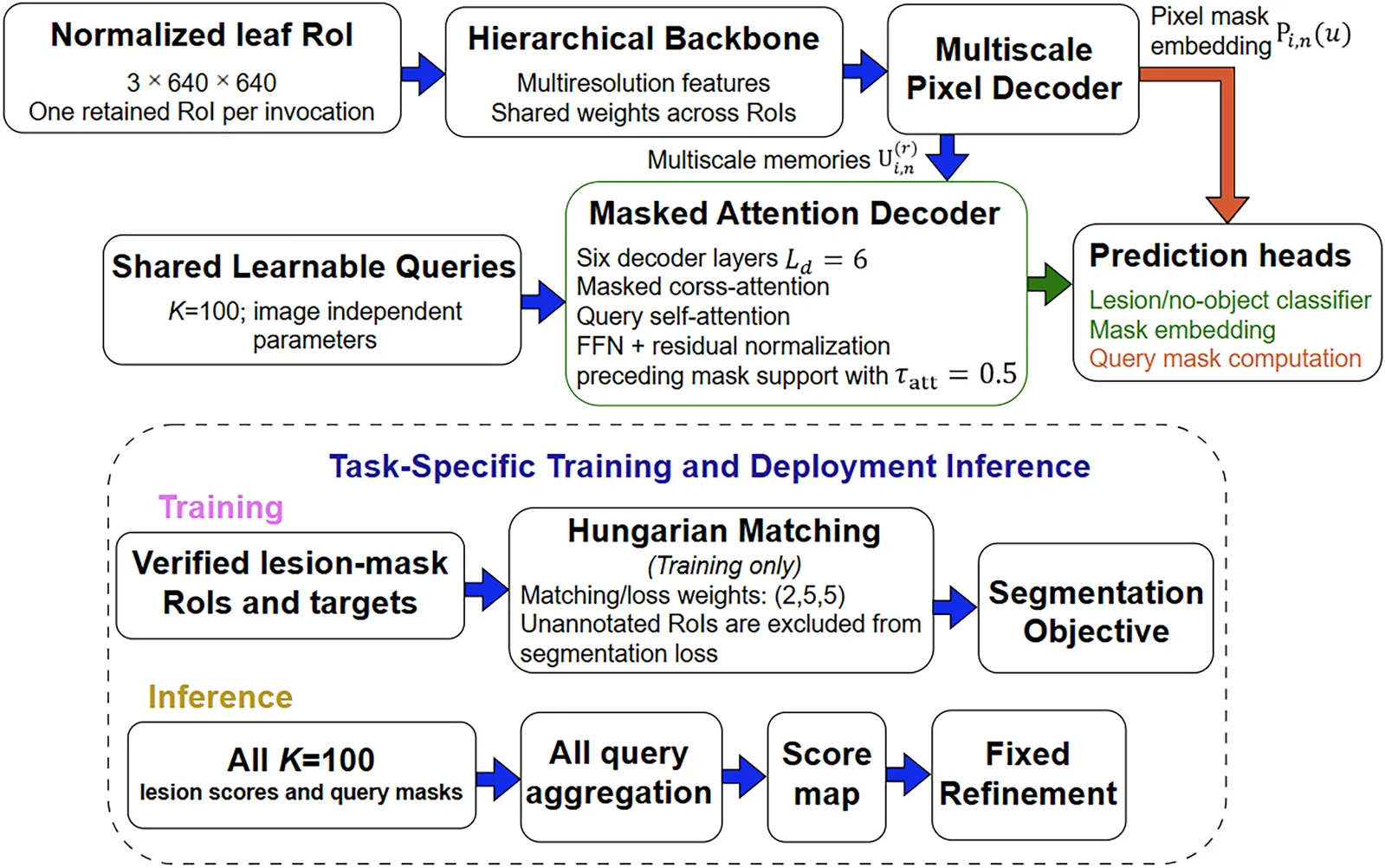
Mask2Former-based binary lesion-segmentation architecture.

The segmentation backbone extracted *L_s_* hierarchical feature levels from each RoI:

(18) $${\left\{{\text{F}}_{i,n}^{\left(r\right)}\right\}}_{r=1}^{L_{s}}=B_{\text{seg}}\left({\text{R}}_{i,n}^{\text{seg}}\right),\;{\text{F}}_{i,n}^{\left(\text{r}\right)}\in {\mathbb{R}}^{C_{r}\times H_{r}\times W_{r}}$$

In Equation 18, $B_{\text{seg}}\left(\cdot \right)$ denotes the Mask2Former image backbone; *r* indexes the backbone feature level; *L_s_* is the number of extracted feature levels; and *C_r_*, *H_r_* and *W_r_* denote the channel dimension, height and width of ${\text{F}}_{i,n}^{\left(\text{r}\right)}$, respectively. The multiscale backbone features were subsequently processed by the pixel decoder as defined in Equation 19:

(19) $$\left({\text{P}}_{i,n}{\left\{{\text{U}}_{i,n}^{\left(r\right)}\right\}}_{r=1}^{L_{d}}\right)=D_{\text{pix}}\left({\left\{{\text{F}}_{i,n}^{\left(\text{r}\right)}\right\}}_{r=1}^{L_{s}}\right)$$

Here, $D_{\text{pix}}\left(\cdot \right)$ denotes the multiscale pixel decoder; ${\text{P}}_{i,n}\in {\mathbb{R}}^{d_{m}\times H_{m}\times W_{m}}$ is the high-resolution per-pixel mask embedding; *d_m_* is its embedding dimension; and ${\text{U}}_{i,n}^{\left(r\right)}$ is the decoder memory associated with feature level *r*. The multiscale memories preserve complementary spatial-resolution and semantic-context information, whereas P*_i_*,*_n_* is used to generate the pixelwise mask logits. The transformer decoder was initialized with a shared bank of *K* learnable query embeddings:

(20) $${\text{Q}}^{\left(0\right)}={\left[q_{1}^{\left(0\right)},\;q_{2}^{\left(0\right)},\;q_{3}^{\left(0\right)},...,q_{K}^{\left(0\right)}\right]}^{\text{T}}\in {\mathbb{R}}^{k\times d_{q}},\;K=100$$

In Equation 20, ${\text{q}}_{k}^{\left(0\right)}\in {\mathbb{R}}^{d_{q}}$ denotes the initial embedding of segmentation query *k*, and *d_q_* is the query-embedding dimension. These initial queries are learnable model parameters shared across all images and RoIs; they are not image-dependent inputs. After interaction with the RoI-specific feature memory, their decoder-layer representations are denoted by ${\text{Q}}_{in}^{\left(l\right)}$. At decoder layer *l*, the multiscale memory level selected for that layer was flattened into *T_l_* feature tokens. Masked cross-attention was then expressed as

(21) $${\text{H}}_{i,n}^{\left(l\right)}=\text{Softmax}\left(\frac{{\tilde{\text{Q}}}_{i,n}^{\left(l-1\right)}{\left({\text{K}}_{i,n}^{\left(l\right)}\right)}^{\text{T}}}{\sqrt{d_{head}}}+{\text{A}}_{i,n}^{\left(l-1\right)}\right){\text{V}}_{i,n}^{\left(l\right)}$$

In Equation 21, ${\tilde{\text{Q}}}_{i,n}^{\left(l-1\right)}\in {\mathbb{R}}^{K\times d_{k}}$ contains the projected query representations from the preceding decoder stage; ${\text{K}}_{i,n}^{\left(l\right)}$, ${\text{V}}_{i,n}^{\left(l\right)}\in {\mathbb{R}}^{T_{l}\times d_{k}}$ are the key and value matrices obtained from the selected multiscale memory; *d_k_* is the key dimension; ${\text{A}}_{i,n}^{\left(l-1\right)}\in {\mathbb{R}}^{K\times T_{l}}$ is the additive attention mask; and ${\text{H}}_{i,n}^{\left(l\right)}$ is the masked cross-attention output. ${\text{K}}_{i,n}^{\left(l\right)}$ denotes the key matrix, whereas *K* denotes the number of segmentation queries.

The permitted attention support for query *k* was obtained by resizing its preceding mask prediction to the spatial resolution of the memory level used at layer *l*. The additive attention mask was defined as

(22) $$\begin{array}{c} A_{i,n,k,t}^{\left(l-1\right)}=\{\begin{array}{c} 0\;\;\;\;\;\;\;t\in \Omega_{i,n,k}^{\left(l-1\right)},\\ -\infty \;\;\;\;\;\;\;others, \end{array}\\ \Omega_{i,n,k}^{\left(l-1\right)}=\left\{t\;:\text{Resiz}{\text{e}}_{r\left(l\right)}\left({\hat{m}}_{i,n,k}^{\left(l-1\right)}\right)\left(t\right)\geq \tau_{\text{att}}\right\},\;\tau_{\text{att}}=0.5 \end{array}$$

In Equation 22, $t\in \left\{1,\ldots .,T_{l}\right\}$ indexes a memory token; ${\hat{m}}_{i,n,k}^{\left(l-1\right)}$ is the mask probability predicted from query *k* at the preceding decoder stage; *r*(*l*) identifies the memory resolution used at layer *l*; and *τ*_all_ is the fixed masked-attention support threshold. For the initial decoder stage, all memory locations were permitted because no preceding mask prediction was available. Each decoder layer additionally contained query self-attention, residual normalization and a feed-forward network; Equation 21 represents only its masked cross-attention operation.

Following the final decoder layer *L_d_* = 6, each query produced a lesion-class probability and a mask embedding. The corresponding prediction heads were

(23) $$\begin{array}{c} p_{i,n,k}^{\text{les}}={\left[\text{Softmax}\left({\text{W}}_{c}{\text{q}}_{i,n,k}^{\left(L_{d}\right)}+{\text{b}}_{c}\right)\right]}_{\text{les}}\\ {\text{e}}_{i,n,k}={\text{W}}_{m}{\text{q}}_{i,n,k}^{\left(L_{d}\right)}+{\text{b}}_{m},\;l_{i,n,k}\left(u\right)=\;{\text{e}}_{i,n,k}^{\text{T}}{\text{P}}_{i,n}\left(u\right),\\ {\hat{m}}_{i,n,k}\left(u\right)=\sigma \left(l_{i,n,k}\left(u\right)\right) \end{array}$$

In Equation 23, ${\text{q}}_{i,n,k}^{\left(L_{d}\right)}$ is the final representation of query *k*; W*_c_* and b*_c_* are the query-classification parameters; $p_{i,n,k}^{\text{les}}$ is the predicted lesion-class probability; and the complementary classifier output represents the no-object class. The parameters W*_m_* and b*_m_* project the query representation to the mask embedding e*_i_*,*_n_*,*_k_*. The vector ${\text{P}}_{i,n}\left(u\right)$ is the pixel embedding at spatial position *u*; *l_i_*,*_n_*,*_k_*(*u*) is the corresponding mask logit; σ(·) is the sigmoid function; and ${\hat{m}}_{i,n,k}\left(u\right)\in \left[0,1\right]$ is the predicted mask probability.

During training, let ${\left\{{\text{g}}_{i,n,j}\right\}}_{j=1}^{J_{i,n}}$ denote the set of verified lesion targets associated with RoI (*i*, *n*). Each annotated RoI had either one lesion-positive union mask $\left(J_{i,n}=1\right)$ or one verified empty mask $\left(J_{i,n}=0\right)$. Separate lesion instances were not treated as independent Mask2Former targets. The *J_i_*,*_n_* verified lesion targets were assigned injectively to the *K* predicted queries using Hungarian matching:

(24) $${\hat{\pi }}_{i,n}=arg\;\underset{\pi \in S_{J_{i,n},K}}{\min}\sum_{j=1}^{J_{i,n}}C_{seg}\left({\text{g}}_{i,n,j},{\hat{\text{s}}}_{i,n,\pi \left(j\right)}\right),\;{\hat{\text{s}}}_{i,n,k}=\left(p_{i,n,k}^{\text{les}},{\hat{m}}_{i,n,k}\right)$$

In Equation 24, $S_{J_{i,n},K}$ denotes the set of injective mappings from the *J_i,n_* verified targets to the *K* segmentation queries; π(*j*) is the query assigned to target *j*; and $\left(p_{i,n,k}^{les},{\hat{m}}_{i,n,k}\right)$ denotes the class-mask prediction generated by query *k*. Hungarian matching was used only to construct the training correspondence and was not part of the deployable inference pathway.

The pairwise matching cost was defined using Equation 25,

(25) $$C_{seg}=\;{\text{λ}}_{\text{ce}}C_{\text{ce}}+{\text{λ}}_{\text{bce}}C_{\text{bce}}+{\text{λ}}_{\text{dice}}C_{\text{dice}}$$

where $C_{\text{ce}}$, $C_{\text{bce}}$ and $C_{\text{dice}}$ are the query-classification, binary-mask and Dice matching costs, respectively.

Segmentation loss was calculated only for RoIs having verified lesion-mask annotations:

(26) $${ℒ}_{\text{seg}}=\;\frac{1}{\left|A_{\text{seg}}\right|}\sum_{\left(i,n\right)\in A_{seg}}\left[{\text{λ}}_{\text{ce}}{ℒ}_{ce}^{\left(i,n\right)}+1\left[J_{i,n}>0\right]\left({\text{λ}}_{\text{bce}}{ℒ}_{bce}^{\left(i,n\right)}+{\text{λ}}_{\text{dice}}{ℒ}_{dice}^{\left(i,n\right)}\right)\right]$$

In Equation 26, $A_{seg}$ is the set of RoIs with verified mask annotations; 1[·] is the indicator function; and ${ℒ}_{ce}^{\left(i,n\right)}$ supervises the lesion/no-object classification of all K queries, with unmatched queries assigned to the no-object class. The binary cross-entropy and Dice losses are averaged over the positively matched target-query pairs. A verified empty mask has $J_{i,n}=0$; therefore, all queries are supervised as no-object and no positive mask-regression term is calculated. RoIs without mask annotations are excluded from $A_{\text{seg}}$ and are not interpreted as lesion-free samples.

For a matched query-target pair containing *P* evaluated pixels, binary cross-entropy was defined as

(27) $${ℒ}_{bce}=\;-\frac{1}{P}\sum_{u=1}^{P}\left[g\left(u\right)\text{log}\left(\hat{m}\left(u\right)+\epsilon \right)+(1-g\left(u\right)\text{log}\left(1-\hat{m}\left(u\right)+\epsilon \right)\right]$$

In Equation 27, (u) indexes a pixel; g(u)∈{0,1} is the verified lesion label; $\hat{m}\left(u\right)\in \left[0,1\right]$ is the corresponding predicted probability; *P* is the number of evaluated pixels; and $\epsilon =1\times 10^{-8}$ is the numerical-stability constant. The indices (*i*, *n*, *j*) are omitted in Equations 27 and 28 only to improve readability.

The corresponding soft Dice loss was

(28) $${ℒ}_{dice}=\;1-\frac{2\sum_{u=1}^{P}\hat{m}\left(u\right)g\left(u\right)+\epsilon }{\sum_{u=1}^{P}\hat{m}\left(u\right)+\sum_{u=1}^{P}g\left(u\right)+\epsilon }$$

Equation 28 measures the differentiable overlap between the predicted lesion probability and its verified target. The smoothing constant *ϵ* prevents division by zero when the predicted or verified positive region is small.

At inference, neither Hungarian matching nor ground-truth masks were available. The final continuous lesion score map was therefore generated by aggregating all *K* query predictions according to their lesion-class probabilities:

(29) $${\text{M}}_{i,n}\left(u\right)=\text{cli}{\text{p}}_{\left[0,1\right]}\left[\sum_{k=1}^{K}p_{i,n,k}^{\text{les}}{\hat{m}}_{i,n,k}\left(u\right)\right]$$

In Equation 29, ${\text{M}}_{i,n}\left(u\right)$ is the continuous lesion score at pixel *u* of retained RoI *n* from image *i*, and ${\text{clip}}_{\left[0,1\right]}\left[\cdot \right]$ restricts the aggregated value to the probability interval. Equation 29 aggregates all inference-time lesion-query predictions; it does not aggregate positively matched training queries. The resulting score map ${\text{M}}_{i,n}\in {\left[0,1\right]}^{640\times 640}$ was forwarded to the fixed lesion-mask refinement procedure described in Section 3.5 and thresholded as defined in Equation 30.

### 3.5 Lesion-mask refinement, region construction and projection

The continuous lesion score map ${\text{M}}_{i,n}\in {\left[0,1\right]}^{640\times 640}$, obtained through the query aggregation defined in Equation 29, was refined using a fixed, non-differentiable post-processing procedure. Here, *i* denotes the input-image index, $n\in {\text{I}}_{i}^{\text{roi}}$ denotes a retained leaf-RoI index and $u\in \Omega_{640}$ denotes a pixel in the 640×640-pixel RoI coordinate system. Ground-truth masks were not used during this inference-time procedure.

The continuous score map was converted into an initial binary lesion mask using the fixed threshold $\tau_{m}=0.5$ specified in Section 4.6:

(30) $${\text{M}}_{i,n}^{b}\left(u\right)=1\left[{\text{M}}_{i,n}\left(u\right)\geq \tau_{m}\right],\;\tau_{m}=0.5,\;u\in \Omega_{640}$$

In Equation 30, 1[·] denotes the indicator function. The threshold was selected exclusively using the PV validation partition and was subsequently frozen for the internal-test and independent-field evaluations A morphological opening followed by closing was applied using the fixed configuration specified in Section 4.6:

(31) $${\text{M}}_{i,n}^{\text{morph}}=\text{Clos}{\text{e}}_{K_{m}}^{\left(n_{c}\right)}\left[\text{Ope}{\text{n}}_{K_{m}}^{\left(n_{o}\right)}\left({\text{M}}_{i,n}^{b}\right)\right]$$

In Equation 31, $K_{m}$ denotes the fixed 3×3 elliptical structuring element, while $n_{o}=1$ and $n_{c}=1$ denote the respective numbers of opening and closing iterations. These settings were applied identically to every retained RoI and were not adjusted separately for the internal-test or field cohorts.

Let ${\left\{C_{i,n,t}\right\}}_{t=1}^{T_{i,n}}$ denote the connected components extracted from ${\text{M}}_{i,n}^{\text{morph}}$, where *T_i,n_* is the number of components identified in RoI *n*. Components smaller than the fixed minimum lesion area ${\text{A}}_{min}=64$ pixels were removed according to Equation 32:

(32) $${\tilde{C}}_{i,n,t}=\{\begin{array}{c} C_{i,n,t},\;\;\;\;\;\left|C_{i,n,t}\right|\geq {\text{A}}_{min},\\ \emptyset ,\;\;\;\;\;\left|C_{i,n,t}\right|<{\text{A}}_{min}, \end{array}\;{\text{A}}_{min}=64$$

The final refined binary lesion mask was obtained by taking the pixelwise union of the retained components, as defined in Equation 33:

(33) $${\text{M}}_{i,n}^{f}\left(u\right)=\overset{t=1}{\underset{T_{i,n}}{\vee }}1\left[u\in {\tilde{C}}_{i,n,t}\right]$$

Thus, ${\text{M}}_{i,n}^{f}\in {\left\{0,1\right\}}^{640\times 640}$, whereas M*i,n* in Equation 29 remains a continuous lesion score map. This distinction prevents the continuous Mask2Former output from being incorrectly described as the final binary mask.

For lesion-positive predictions, the refined mask was broadcast across the three RGB channels and applied to the enhanced RoI ${\text{E}}_{i,n}^{640}$ defined in Equation 17. When no lesion component survived the fixed refinement procedure, the complete enhanced leaf RoI was retained:

(34) $${\text{E}}_{i,n}^{\text{iso}}=\{\begin{array}{cc} {\text{E}}_{i,n}^{640}⊙{\text{M}}_{i,n}^{f} & \sum_{u\in {Ω}_{640}}{\text{M}}_{i,n}^{f}\left(u\right)>0,\\ {\text{E}}_{i,n}^{640} & \sum_{u\in {Ω}_{640}}{\text{M}}_{i,n}^{f}\left(u\right)=0 \end{array}$$

In Equation 34, ⊙ denotes channel-wise element multiplication and ${\text{E}}_{i,n}^{\text{iso}}$ denotes the lesion-focused RGB representation. The deterministic fallback prevents verified healthy leaves and lesion false-negative cases from being converted into uninformative all-zero tensors. It does not introduce a ground-truth lesion mask or replace the predicted mask.

For image-coordinate segmentation evaluation and visualization, the refined RoI mask was projected back to its expanded position in the original image using the geometric transformation ${\text{Φ}}_{i,n}$ retained during RoI extraction:

(35) $${\text{M}}_{i,n}^{\text{img}}=\text{Projec}{\text{t}}_{{\text{Φ}}_{i,n}}\left({\text{M}}_{i,n}^{f}\right)\text{ and }{\text{M}}_{i}^{\text{img}}\left(v\right)=\underset{n\in {\text{I}}_{i}^{\text{roi}}\;}{\max}{\text{M}}_{i,n}^{\text{img}}\left(v\right),\;v\in {Ω}_{i}^{\text{img}}$$

As expressed by Equation 35, ${\text{Φ}}_{i,n}$ performs nearest-neighbor mask resizing to the dimensions of the expanded bounding box $b_{i,n}^{\delta }$ and places the resized mask at the corresponding location in the original *H_i_*×*W_i_*-pixel image. When multiple retained RoIs overlap, their projected masks are combined using the pixelwise maximum. Images without a retained RoI produce an empty projected mask and remain recorded as end-to-end localization failures.

Only the projected masks ${\text{M}}_{i}^{\text{img}}$, rather than resized RoI-coordinate masks, were used for image-coordinate segmentation evaluation. The projected masks were evaluated using the fixed threshold and post-processing configuration defined in Section 4.6. Lesion-positive and verified empty-mask cases were evaluated separately.

### 3.6 EfficientNet-B4 feature extraction and channel-attention refinement

The lesion-focused RGB representation ${\text{E}}_{i,n}^{640}$ defined in Equation 34 is suitable for visualization of the isolated disease region. However, the tensor supplied to EfficientNet-B4 was constructed in normalized feature space so that pixels excluded by the lesion mask had a numerical value of zero. Normalizing an already masked RGB image would instead transform black background pixels into nonzero channel-normalized values.

Let ${\text{R}}_{i,n}^{\text{cls}}\in {\mathbb{R}}^{3\times 380\times 380}$ denote the normalized classification-branch tensor generated during the branch-specific RoI preparation defined in Equation 17. The refined 640×640-pixel binary mask ${\text{M}}_{i,n}^{f}$ was resized to the EfficientNet-B4 input resolution using nearest-neighbor interpolation. The operational classifier input was then constructed as

(36) $$\begin{array}{c} {\text{M}}_{i,n}^{\text{cls}}=\;\text{Resiz}{\text{e}}_{\text{nearest}}\left({\text{M}}_{i,n}^{f},\left(380,380\right)\right),\\ {\text{X}}_{i,n}=\{\begin{array}{cc} {\text{R}}_{i,n}^{\text{cls}}⊙{\text{M}}_{i,n}^{\text{cls}}, & \sum_{u\in {Ω}_{380}}{\text{M}}_{i,n}^{\text{cls}}\left(u\right)>0,\\ {\text{R}}_{i,n}^{\text{cls}}, & \sum_{u\in {Ω}_{380}}{\text{M}}_{i,n}^{\text{cls}}\left(u\right)=0 \end{array} \end{array}$$

In Equation 36, ${\text{M}}_{i,n}^{\text{cls}}\in {\left\{0,1\right\}}^{380\times 380}$ is broadcast across the three channels before multiplication. Because ${\text{R}}_{i,n}^{\text{cls}}$ was normalized before mask application, excluded pixels remained zero in normalized feature space. The deterministic second branch retained the complete normalized leaf RoI for verified healthy cases and for cases in which no predicted lesion survived the fixed post-processing defined in Equations 30–33. No ground-truth mask was substituted in this fallback branch.

EfficientNet uses compound scaling to jointly control network depth, channel width and input resolution. To avoid reusing *r*, which denotes the multiscale feature-level index in Equations 18 and 19, the scaled quantities are represented by *d*_net_, *w*_net_ and *s*_in_ Equation 37:

(37) $$d_{\text{net}}=\alpha^{\phi },\;w_{\text{net}}=\beta^{\phi },\;s_{\text{in}}=\gamma^{\phi }$$

The scaling coefficients satisfy Equation 38.

(38) $$\alpha \beta^{2}\gamma^{2}\approx 2,\;\alpha \geq 1,\;\beta \geq 1,\;\gamma \geq 1$$

The EfficientNet-B4 configuration corresponds to *ϕ* = 4 and uses an input resolution of 380×380 pixels. Its compound-scaled backbone structure was retained without architectural modification. The final convolutional feature map for retained RoI *n* of image *i* was obtained as defined in Equation 39.

(39) $${\text{F}}_{i,n}^{\text{B}4}=\epsilon_{\text{B}4}\left({\text{X}}_{i,n}\right)$$

where $\epsilon_{B4}\left(\cdot \right)$ denotes the EfficientNet-B4 convolutional backbone. The resulting feature map satisfies

(40) $${\text{F}}_{i,n}^{\text{B}4}\in {\mathbb{R}}^{C\times H_{f}\times W_{f}},\;C=1792$$

In Equation 40, *C* is the terminal channel dimension, while *H_f_* and *W_f_* denote the spatial dimensions of the final convolutional representation. These spatial dimensions are distinct from the original-image dimensions *H_i_* and *W_i_* used in Equation 35. Global average pooling converted the convolutional feature map into a fixed-dimensional RoI representation using Equation 41:

(41) $${\text{z}}_{i,n,c}=\frac{1}{H_{f}W_{f}}\sum_{x=1}^{H_{f}}\sum_{y=1}^{W_{f}}{\text{F}}_{ii,n}^{\text{B}4}\left(c,x,y\right)\;c=1,\ldots .,C$$

The corresponding pooled feature vector was calculated as defined in Equation 42

(42) $${\text{z}}_{i,n}=\;{\left[{\text{z}}_{i,n,1},{\text{z}}_{i,n,2},\ldots .,{\text{z}}_{i,n,C}\right]}^{\text{T}}\in {\mathbb{R}}^{C}$$

A channel-attention refinement layer was applied to the pooled representation to learn the relative contribution of each feature channel. The channel-gating vector was calculated using Equation 43.

(43) $${\text{a}}_{i,n}=\sigma \left[{\text{W}}_{2}\text{ReLU}\left({\text{W}}_{1}{\text{z}}_{i,n}+{\text{b}}_{1}\right)+{\text{b}}_{2}\right]$$

where ${\text{W}}_{1}\in {\mathbb{R}}^{\left(C/r_{c}\right)\times C}$, ${\text{W}}_{2}\in {\mathbb{R}}^{C\times \left(C/r_{c}\right)}$ and *r_c_* = 16. For *C* = 1792 and *r_c_* = 16, the intermediate channel-attention dimension is $C/r_{c}=112$. The sigmoid function σ[·] constrains each element of a*_i,n_* to the interval (0,1). The attention-refined RoI feature representation was obtained through channel-wise multiplication using Equation 44:

(44) $${\tilde{z}}_{i,n}={\text{a}}_{i,n}⊙{\text{z}}_{i,n}$$

During training, dropout with probability *p_drop_* = 0.5 was applied to the attention-refined representation Equation 45:

(45) $${\text{d}}_{i,n}=\text{Dropout}\left({\tilde{z}}_{i,n};p_{drop}=0.5\right)$$

Dropout was disabled during validation and final inference. The resulting representation ${\tilde{z}}_{i,n}\in {\mathbb{R}}^{1792}$ was supplied to the complete 38-category classification head described in Section 3.7. The same EfficientNet-B4 and channel-attention parameters were reused for every retained RoI; therefore, processing multiple RoIs increased the number of forward invocations but did not increase the model parameter count.

### 3.7 Disease classification and multi-RoI aggregation

During training, the dropout-regularized representation d*_i,n_* was supplied to the classification head. During validation and inference, dropout was disabled and the attention-refined representation ${\tilde{z}}_{i,n}\in {\mathbb{R}}^{1792}$ was supplied directly. Here, *i* denotes the input-image index and $\text{n}\in {\text{I}}_{i}^{\text{roi}}$ denotes a retained leaf-RoI index. The same classification-head parameters were shared across all retained RoIs.

A fully connected layer projected the 1792-dimensional EfficientNet-B4 representation into a $d_{h}=512$-dimensional hidden space as in Equation 46:

(46) $${\text{u}}_{i,n}={\text{W}}_{h}{\text{d}}_{i,n}+{\text{b}}_{h},\;{\text{W}}_{h}\in {\mathbb{R}}^{512\times 1792},\;{\text{b}}_{h}\in {\mathbb{R}}^{512}$$

Batch normalization and ReLU activation were subsequently applied:

(47) $${\hat{u}}_{i,n,j}=\frac{u_{i,n,j}-\mu_{ℬ,j}}{\sqrt{\sigma_{ℬ,j}^{2}+\epsilon_{BN}}},\;{\text{h}}_{i,n}=\text{ReLU}\left(\gamma ⊙{\hat{u}}_{i,n}+\beta \right)$$

In Equation 47, $\mu_{ℬ,j}$ and $\sigma_{ℬ,j}^{2}$ are the mini-batch mean and variance of hidden feature *j*, *ϵ_BN_* is the batch-normalization stability constant, and *γ* and *β* are learnable scale and shift parameters. Running statistics accumulated during training were used during validation and final inference. The classifier retained the complete $C_{\text{cls}}=38$-category output space. The RoI-level classification logits were calculated using Equation 48:

(48) $${\text{o}}_{i,n}={\text{W}}_{o}{\text{h}}_{i,n}+{\text{b}}_{o},\;{\text{W}}_{o}\in {\mathbb{R}}^{38\times 512},\;{\text{b}}_{o}\in {\mathbb{R}}^{38},\;{\text{o}}_{i,n}\in {\mathbb{R}}^{38}$$

The corresponding RoI-level softmax probability for category *c* was

(49) $$p_{i,n,c}^{\text{cls}}=\frac{exp\left({\text{o}}_{i,n,c}\right)}{\sum_{q=1}^{C_{cls}}exp\left({\text{o}}_{i,n,q}\right)},\;c\in \left\{1,\ldots .,C_{cls}\right\},\;C_{cls}=38$$

The class-index variable *q* in Equation 49 is used only for softmax normalization and is distinct from the segmentation-query index *k* used in Equations 20–29. When image *i* contained more than one retained RoI, the RoI-level probability vectors were combined using arithmetic-mean aggregation:

(50) $$p_{i,c}^{\text{img}}=\frac{1}{N_{i}^{\text{roi}}}\sum_{\text{n}\in {\text{I}}_{i}^{\text{roi}}}p_{i,n,c}^{\text{cls}}\;,\;N_{i}^{\text{roi}}>0$$

As defined in Equation 50, every retained RoI contributed equally to the final image-level probability vector. This aggregation increased the number of classifier invocations when multiple leaves were retained but did not duplicate the EfficientNet-B4 or classification-head parameters.

The image-level disease prediction was obtained using Equation 51

(51) $${\hat{y}}_{i}=arg\underset{c\in \left\{1,\ldots .,C_{cls}\right\}}{\max}p_{i,c}^{\text{img}}$$

and the associated softmax confidence score was

(52) $$s_{i}^{\text{cls}}=\underset{c\in \left\{1,\ldots .,C_{cls}\right\}}{\max}p_{i,c}^{\text{img}}$$

The quantity $s_{i}^{\text{cls}}$ in Equation 52 is reported as a softmax confidence score. It must not be interpreted as a calibrated posterior probability unless a separate probability-calibration procedure is explicitly applied and evaluated. If no RT-DETR prediction satisfied the frozen RoI-retention rule, EfficientNet-B4 was not invoked. No ground-truth box or manually selected leaf crop was substituted in the deployable configuration. The corresponding image was retained as an end-to-end localization failure rather than being assigned an artificial classification result.

For classification training, the target vector was defined as

(53) $${\text{y}}_{i}^{\text{tar}}=\{\begin{array}{cc} \text{O}\text{n}\text{e}\text{H}\text{o}\text{t}\left(y_{i}\right), & Ordinary\;training\;sample,\\ {\text{λ}}_{i}y_{i}^{\left(a\right)}+\left(1-{\text{λ}}_{i}\right)y_{i}^{\left(b\right)} & MixUp\;training\;sample, \end{array}\;\sum_{c=1}^{C_{\text{cls}}}{\text{y}}_{i,c}^{\text{tar}}=1$$

In Equation 53, ${\text{λ}}_{i}\in \left[0,1\right]$ is the MixUp coefficient, while ${\text{y}}_{i}^{\left(a\right)}$ and ${\text{y}}_{i}^{\left(b\right)}$ are the target vectors of the two source images. Thus, ${\text{y}}_{i,c}\in \left\{0,1\right\}$ for an ordinary sample and ${\text{y}}_{i}^{\left(c\right)}\in \left[0,1\right]$ for a MixUp sample. The classification objective was categorical cross-entropy calculated over images having verified image-level labels and valid classifier inputs:

(54) $${ℒ}_{\text{cls}}=-\frac{1}{\left|A_{\text{cls}}\right|}\sum_{i\in A_{\text{cls}}}\sum_{c=1}^{C_{\text{cls}}}{\text{y}}_{i,c}^{\text{tar}}\text{log}\left(p_{i,c}^{img}\right)$$

In Equation 54, $A_{\text{cls}}$ denotes the set of training images containing verified image-level labels for which at least one valid classification RoI was available. Missing image-level labels were excluded from the classification objective and were not converted into negative targets.

The complete 38-category classifier was retained during the PV internal-test and independent-field evaluations. A separate nine-category classifier was not trained for the source-matched analysis. When evaluating the common nine-category subset, predictions assigned to any of the remaining 29 PV categories were counted as classification errors; they were not removed and the corresponding probability vector was not renormalized. The classification loss defined in Equation 54 was subsequently combined with the applicable detection and segmentation objectives through the adaptive multi-task weighting procedure described in Section 3.8.

### 3.8 Adaptive task-loss weighting and AdamW optimization

The coordinated training schedule included detection, segmentation and classification objectives. Because their numerical scales and convergence behaviors differed, EMA normalization and smoothed adaptive weighting were applied before the AdamW parameter update.

Let $T_{t}\subseteq \left\{\text{det},\text{ seg},\text{ cls}\right\}$ denote the set of tasks having valid supervision at training iteration *t*, and $K_{t}=\left|T_{t}\right|$. Detection was active only when the current samples contained verified bounding-box annotations. Segmentation was active for samples containing verified lesion masks, including verified empty masks, whereas samples without mask annotations were excluded. Classification was active for samples having verified image-level labels and at least one valid classifier input. Missing annotations were not converted into negative targets.

In this subsection, $k\in T_{t}$ denotes a task label and is distinct from the segmentation-query index *k* used in Equations 20–29. For active task *k*, the EMA loss statistic was updated as

(55) $$s_{k}^{\left(t\right)}=\rho s_{k}^{\left(t-1\right)}+\left(1-\rho \right)\text{sg}\left({ℒ}_{k}^{\left(t\right)}\right),\;\rho =0.9,\;k\in T_{t}$$

In Equation 55, sg(·) denotes a stop-gradient operation. When task *k* was encountered for the first time, $s_{k}^{\left(t\right)}$ was initialized using its detached task loss. When $k\notin T_{t}$, its stored EMA statistic remained unchanged. The active task loss was normalized using its corresponding EMA statistic:

(56) $${\tilde{ℒ}}_{k}^{\left(t\right)}=\frac{{ℒ}_{k}^{t}}{s_{k}^{\left(t\right)}+\epsilon }\;,\;\epsilon =1\times 10^{-8},\;k\in T_{t}$$

As defined in Equation 56, the EMA statistic normalizes the magnitude of each objective without introducing gradient propagation through its historical loss values. The instantaneous weight of each active task was calculated using temperature-scaled softmax normalization:

(57) $${\hat{w}}_{k}^{\left(t\right)}=K_{t}\frac{\text{exp}\left[\text{sg}\left({\tilde{ℒ}}_{k}^{\left(t\right)}\right)/T\right]}{\sum_{j\in \tau_{t}}\text{exp}\left[\text{sg}\left({\tilde{ℒ}}_{j}^{\left(t\right)}\right)/T\right]}\;,\;\tau =2.0,\;k\in T_{t}$$

The factor *K_t_* in Equation 57 maintains an average active-task weight of one across the active tasks. Thus, changes in the number of active supervised tasks do not arbitrarily reduce the scale of the coordinated objective.

To suppress iteration-to-iteration fluctuations, the instantaneous weights were exponentially smoothed and subsequently renormalized over the active tasks:

(58) $$\begin{array}{c} q_{k}^{\left(t\right)}=\eta q_{k}^{\left(t-1\right)}+\left(1-\eta \right){\hat{w}}_{k}^{\left(t\right)},\;k\in T_{t}\\ w_{k}^{\left(t\right)}=K_{t}\frac{q_{k}^{\left(t\right)}}{\sum_{j\in T_{t}}q_{j}^{\left(t\right)}},\;\eta =0.9,\;q_{k}^{\left(0\right)}=1 \end{array}$$

For an inactive task $k\notin T_{t}$, $q_{k}^{\left(t\right)}$ retained its previously stored value. The renormalization in Equation 58 ensures that $\sum_{k\in T_{t}}w_{k}^{\left(t\right)}=K_{t}$. The adaptively weighted multi-task objective is defined in Equation 59

(59) $${ℒ}_{\text{coord}}^{\left(t\right)}=\sum_{k\in T_{t}}sg\left(w_{k}^{\left(t\right)}\right){\tilde{ℒ}}_{k}^{\left(t\right)}$$

The stop-gradient operations in Equations 55, 57, and 59 prevent gradients from propagating through the EMA statistics and adaptive-weight calculations. For each active task, gradients are propagated only through the corresponding normalized task loss to its associated module parameter block; no gradients cross module boundaries. EMA normalization and adaptive weighting modify only the scalar coefficients applied to the task losses before optimization. The resulting task-specific gradients were applied using the standard AdamW optimizer.

This formulation does not create differentiable paths through detector-confidence filtering, RoI retention, crop extraction, mask thresholding, morphological refinement, connected-component filtering or multi-RoI selection. Accordingly, RT-DETR is optimized through ${ℒ}_{\text{det}}$, Mask2Former through ${ℒ}_{\text{seg}}$, and EfficientNet-B4 through ${ℒ}_{\text{cls}}$. The term coordinated multi-module training therefore denotes coordinated optimization of the task-specific objectives within a common schedule, rather than fully differentiable end-to-end propagation through every pipeline operation.

Let *θ*_det_, *θ*_seg_, and *θ*_cls_ denote the disjoint trainable parameter sets of RT-DETR, Mask2Former, and EfficientNet-B4, respectively, and let $\theta =\text{Concat}\left(\theta_{\text{det}},\theta_{\text{seg}},\theta_{\text{cls}}\right)$ denote the complete trainable parameter vector. The inter-module pathway contains hard, gradient-detached operations, including confidence-based RoI selection, discrete crop extraction from predicted boxes, binary-mask thresholding, morphological refinement, connected-component filtering, and lesion-region construction. Consequently, downstream task losses do not establish differentiable computational paths across module boundaries, and each module receives new supervised gradient contributions only from its corresponding task objective. For k∈{det, seg, cls}, the module-specific gradient block and assembled task-loss gradient at iteration t are defined by Equation 60.

(60) $$\begin{array}{c} {\text{g}}_{t,k}=\{\begin{array}{cc} {\text{sg}\left(w_{k}^{\left(t\right)}\right)\nabla_{\theta_{k}}{\tilde{ℒ}}_{k}^{\left(t\right)}|}_{\theta_{k}=\theta_{k,t-1}}, & k\in T_{t}\\ 0, & k\notin T_{t} \end{array}\\ \nabla_{\theta_{j}}{\tilde{ℒ}}_{k}^{\left(t\right)}=0,\;j\neq k,\;k\in T_{t}\\ {\text{g}}_{t}=\text{Concat}\left({\text{g}}_{t,\text{det}},\;{\text{g}}_{t,\text{seg}},\;{\text{g}}_{t,\text{cls}}\right) \end{array}$$

where g*_t,k_* denotes the loss-gradient contribution associated with parameter block *θ_k_*. An inactive task contributes no new supervised gradient at iteration *t*. The condition $\nabla_{\theta_{j}}{\tilde{ℒ}}_{k}^{\left(t\right)}=0$ for j≠k, explicitly states that the supervised objective of task *k* does not backpropagate through the hard inter-module interfaces to another module. The assembled gradient g*_t_* represents the task-loss gradient subsequently used by the AdamW optimization step. Hence, the EMA-normalized adaptive weighting mechanism modulates the relative magnitude of task-specific gradient contributions without introducing cross-module gradient propagation or shared-parameter learning. Accordingly, coordinated multi-module training denotes synchronized optimization of separately supervised modules within a common training schedule.

AdamW accumulated the first- and second-moment estimates as defined in Equation 61

(61) $${\text{M}}_{t}=\beta_{1}{\text{M}}_{t-1}+\left(1-\beta_{1}\right){\text{g}}_{t},\;{\text{v}}_{t}=\beta_{2}{\text{v}}_{t-1}+\left(1-\beta_{2}\right)\left({\text{g}}_{t}⊙{\text{g}}_{t}\right)$$

The bias-corrected moment estimates were calculated using Equation 62:

(62) $${\hat{m}}_{t}=\frac{{\text{M}}_{t}}{1-\beta_{1}^{t}}\;,\;{\hat{v}}_{t}=\frac{{\text{v}}_{t}}{1-\beta_{2}^{t}}$$

Using decoupled weight decay, the AdamW parameter update was performed according to Equation 63:

(63) $${\text{θ}}_{t}={\text{θ}}_{t-1}-\alpha_{t}\frac{{\hat{m}}_{t}}{\sqrt{{\hat{v}}_{t}}+\epsilon }-\alpha_{t}{\text{λ}}_{wd}{\text{θ}}_{t-1}\;,\;{\text{λ}}_{wd}=1\times 10^{-4}$$

The learning rate followed cosine annealing:

(64) $$\alpha_{t}=\alpha_{\text{min}}+\frac{1}{2}\left(\alpha_{\text{max}}-\alpha_{\text{min}}\right)\left[1+\text{cos}\left(\frac{\pi t}{T_{max}}\right)\right]\;,\;0\leq t\leq T_{max},\;\alpha_{\text{max}}=10^{-4},\;\alpha_{\text{min}}=10^{-6}$$

In Equation 64, *T_max_* denotes the maximum number of optimizer iterations corresponding to the 150-epoch training budget. Early stopping could terminate optimization before iteration *T_max_* when the mean EMA-normalized PV validation loss defined in Section 4.3 failed to decrease by more than 1×10^−4^ for 20 consecutive epochs.

Adaptively weighted AdamW training therefore does not replace the internal AdamW update rule. It applies EMA-based task-loss normalization and smoothed adaptive weighting before the standard AdamW parameter update. Its empirical contribution was evaluated through the controlled fixed-weight and optimizer comparisons reported in **Table 3**, **4**.

**Table 3:** Comparison of training schedules and task-loss-weighting strategies under stage-specific gradient updates.

Training strategy	PV AP@0.5 (%)	PV Dice (%)	Matched-PV macro-F1 (%)	Field AP@0.5 (%)	Field dice (%)	Field macro-F1 (%)
Sequential module-wise training with frozen upstream checkpoints	92.95	88.92	94.96	85.89	71.77	76.80
Coordinated schedule with stage-specific gradients and fixed equal task-loss weights	94.95	90.92	95.97	87.89	74.78	78.81
Coordinated schedule with stage-specific gradients and EMA-normalized adaptive task-loss weights	95.63	92.47	96.77	89.28	77.90	80.80

**Table 4:** Comparison of optimization and loss-weighting strategies.

Base optimizer & Task weighting	Task weighting	PV AP@ 0.5 (%)	PV Dice (%)	Matched PV-9 macro-F1 (%)	Field AP@0.5 (%)	Field Dice (%)	Field-9 macro-F1 (%)	Convergence epoch
SGD	EMA-normalized, Fixed ( $\omega_{k}=1$)	90.94	84.91	91.95	80.86	60.73	68.77	118
Adam		92.95	87.91	93.96	83.88	67.76	73.79	96
AdamW		94.95	90.92	95.97	87.89	74.78	78.81	89
AdamW + adaptive task-loss weighting	EMA-normalized, adaptive ( $\omega_{k}^{\left(t\right)}$)	95.63	92.47	96.77	89.28	77.90	80.80	72

## 4 Experimental setup

This section describes the data sources, leakage-control procedure, annotation protocol, implementation environment, training configuration, evaluation design and computational-complexity analysis used for the proposed RT-DETR–Mask2Former framework. The experimental design separates controlled-domain model development from independent field evaluation and reports classification, class-agnostic leaf localization, lesion segmentation and computational efficiency using task-specific annotation subsets and evaluation units.

### 4.1 Dataset sources, composition and leakage-controlled partitioning

The study used the publicly available PV dataset as the controlled-domain source and an independently acquired field cohort as the external evaluation source. The dataset composition, leakage-control grouping, and annotation coverage for both sources are summarized in **Table 5**. PV contained 54,306 RGB images representing 38 healthy and diseased host–condition categories across 14 crop species. The independent field cohort contained 4,200 RGB images from 125 plants acquired over 10 imaging sessions and represented nine categories mapped to categories available in PV.

**Table 5:** Source-separated dataset composition, leakage-control groups and annotation coverage.

Dataset/Task subset	Categories	Unique images	Annotation records	Leakage-control unit
PV training partition (70%)	38	38,014	38,014 image labels	14,011 leaf-identity/duplicate clusters
PV validation partition (15%)	38	8,146	8,146 image labels	3,002 leaf-identity/duplicate clusters
PV internal-test partition (15%)	38	8,146	8,146 image labels	3,002 leaf-identity/duplicate clusters
Total PV source	38	54,306	54,306 image labels	20,015 leaf-identity/duplicate clusters
Independent field cohort	9 shared categories	4,200	4,200 image labels	125 plants across 10 acquisition sessions
PV detection-annotated subset	9	10,760	13,450 leaf bounding boxes	≈ 3,966 inherited PV leaf-identity/duplicate clusters
Field detection-annotated subset	9	4,200	5,250 leaf bounding boxes	125 plant-level clusters
Complete detection annotation inventory	9	14,960	18,700 leaf bounding boxes	≈ 4,091 Source-specific groups: 3,966 PV groups and 125 field-plant clusters
PV fully annotated segmentation/multitask subset	9	5,720	7,150 leaf-level mask records	≈ 2,108 inherited PV leaf/duplicate groups
Field fully annotated segmentation/multitask subset	9	4,200	5,250 leaf-level mask records	125 plant-level clusters
Complete fully annotated segmentation/multitask inventory	9	9,920	12,400 leaf-level mask records	≈ 2,233 source-specific groups: 2,108 PV groups and 125 field-plant clusters
Complete image inventory	38 overall; 9 represented in the field cohort	58,506	58,506 image labels	20,140 source-specific groups: 20,015 PV groups and 125 field-plant clusters

PV was the only source used for model development. After duplicate grouping, 38,014 images were assigned to training, 8,146 to validation and 8,146 to the fixed internal-test partition. The field cohort was reserved in its entirety for independent evaluation and was not used for model fitting, augmentation design, early stopping, checkpoint selection, hyperparameter tuning, threshold calibration or post-processing selection. PV internal-test and field results were calculated separately and were not pooled.

Each sample was represented using the unified annotation format using Equation 65,

(65) $$D={\left\{\left(I_{i},B_{i},M_{i},Y_{i},s_{i},g_{i}\right)\right\}}_{i=1}^{N}$$

where *I_i_* denotes the RGB image, *B_i_* represents the set of leaf bounding-box annotations, *M_i_* denotes the pixel-level lesion masks, *Y_i_* is the corresponding image-level class label, *N* is the total number of images, *s_i_* identifies the data source and *g_i_* identifies the leakage-control group. When a sample was not annotated for a particular spatial task, the corresponding value was recorded as unavailable rather than interpreted as a negative target. For segmentation, $M_{i}=\emptyset$ denotes an unavailable mask annotation, whereas $M_{i}=0$ denotes a verified lesion-free or empty mask. This distinction was retained during both training and evaluation.

The detection annotation subset contained 14,960 unique images and 18,700 leaf bounding-box instances. This inventory comprised 10,760 PV images with 13,450 bounding boxes and 4,200 field images with 5,250 bounding boxes. The fully annotated segmentation/multitask inventory contained 9,920 images and 12,400 leaf-level mask records: 5,720 PV images with 7,150 mask records and 4,200 field images with 5,250 mask records. Each image in the fully annotated subset had an image-level class label and corresponding leaf boxes and mask records. The segmentation subset was therefore nested within the detection subset and should not be added to it when calculating the number of unique images.

Before partitioning, exact duplicate files were identified using SHA-256 hashes. Source identifiers, where available, and candidate near-duplicate pairs identified using 64-bit DCT-based perceptual hashes with a Hamming distance not exceeding five were subsequently inspected. Confirmed duplicate or repeated-leaf images were consolidated into mutually exclusive leaf/specimen groups. Group-level stratification with random seed 42 produced 14,011 training groups, 3,002 validation groups and 3,002 internal-test groups. Augmented derivatives were generated only after partitioning and remained associated with their source training group.

For the independent field cohort, all images belonging to the same plant were retained within a common evaluation cluster, including images acquired from that plant during different sessions. Cluster-bootstrap confidence intervals were calculated by resampling the 125 plant-level clusters and retaining all images associated with each selected plant. No field image or field plant-level cluster contributed to the PV training, validation or internal-test partitions. Group-aware five-fold cross-validation was conducted only on the 46,160-image PV development cohort formed by the fixed training and validation partitions; the internal-test and field cohorts remained excluded.

### 4.2 Annotation protocol and quality control

Image-level labels, leaf bounding boxes and lesion masks were maintained as separate annotation layers. Image-level labels defined the 38-category classification target. Bounding boxes and masks were available only for the nine-category spatially annotated subset. RT-DETR was trained as a class-agnostic leaf localizer; disease-category prediction was performed downstream by the lesion-guided EfficientNet-B4 classifier.

Each visible and sufficiently separable target leaf was assigned an instance identifier and a bounding box enclosing its visible extent while limiting unnecessary background. Images containing multiple separable leaves could therefore contribute more than one bounding-box instance. Boxes were clipped to the image boundaries, and degenerate or zero-area boxes were rejected. When overlapping leaves could not be separated reliably, the instance was flagged for manual review rather than treated automatically as a valid detection target.

A leaf-level mask record was associated with each fully annotated leaf instance. Lesion-positive masks delineated visible symptom-bearing tissue, including spots, necrosis, chlorosis, mildew and irregular lesion regions. Verified healthy or lesion-free leaves were represented by all-zero masks and retained for the empty-mask false-positive analysis. Images with a reliable image-level diagnosis but without sufficiently visible or reproducible lesion boundaries could remain in the classification dataset while being excluded from lesion-positive mask-overlap evaluation. Consequently, an unavailable mask and a verified empty mask represented different annotation states.

Quality control included verification of box coverage, background inclusion, correspondence between box and mask instance identifiers, lesion-boundary consistency, mask projection validity and empty-mask status. Ambiguous cases involving overlapping foliage, severe blur, weak symptoms, low contrast or uncertain lesion boundaries were subjected to manual review. An annotation was accepted for segmentation only when the visible lesion boundary could be delineated consistently; otherwise, the spatial annotation was excluded without discarding an otherwise reliable image-level label.

The final annotation manifest recorded the image identifier, source, host–condition label, leakage-control group, annotation-availability flags, bounding-box instance identifiers, mask identifiers and lesion-positive/empty-mask status. The same source-separated manifests were used for the proposed framework, reimplemented baselines, ablations and oracle controls. This ensured that differences among methods were not caused by changes in annotation availability or evaluation membership.

### 4.3 Training strategy and data processing

Model fitting was restricted to the leakage-controlled PV training partition. The PV validation partition was used only for early stopping, checkpoint selection and calibration of inference thresholds and post-processing settings. The fixed PV internal-test partition and independent field cohort were excluded from all model-development decisions. Stochastic transformations were applied only during training, whereas PV validation, PV internal-test and field samples underwent deterministic preprocessing.

Classification supervision was activated for samples with verified image-level labels and at least one valid classifier input. Detection and segmentation supervision were activated only for samples containing verified leaf boxes and lesion masks, respectively. Missing spatial annotations were recorded as unavailable and were not converted into negative targets. RT-DETR predictions and Mask2Former outputs were detached before downstream hard processing, and each task loss updated only its associated module parameter block.

Training was limited to 150 epochs. Validation was performed once after every epoch on the fixed PV validation partitions with stochastic augmentation disabled. The checkpoint-selection criterion was the mean of the EMA-normalized detection, segmentation and classification validation losses as defined in Equation 66,

(66) $$V^{\left(e\right)}=\frac{1}{3}\left({\tilde{ℒ}}_{\text{det},\text{val}}^{\left(e\right)}+{\tilde{ℒ}}_{\text{seg},\text{val}}^{\left(e\right)}+{\tilde{ℒ}}_{\text{cls},\text{val}}^{\left(e\right)}\right),$$

The training-derived EMA statistics were held fixed and were not updated using validation samples. Lower values of $V^{\left(e\right)}$ indicated better validation performance. A decrease greater than 1×10^−4^ relative to the best previously recorded value was treated as an improvement. When two checkpoints differed by no more than this tolerance, the earlier checkpoint was retained. Training stopped after 20 consecutive epochs without a qualifying improvement. The same selection metric, validation frequency, improvement tolerance, epoch budget and stopping rule were used for the training-strategy and optimizer comparisons reported in **Table 3**, **4**.

After checkpoint selection, the inference thresholds and post-processing settings were calibrated and frozen using the validation-only procedure described in Section 4.6. Ground-truth RoIs and lesion masks were used only in the explicitly identified oracle controls reported in **Table 1**.

### 4.4 Implementation environment

The framework was implemented using Python 3.10.12, PyTorch 2.1.2 and torchvision 0.16.2 under Ubuntu 22.04 LTS. GPU execution used CUDA 12.1, cuDNN 8.9 and NVIDIA driver 535.104.05 on an NVIDIA RTX 4090 GPU with 24 GB VRAM. Automatic mixed-precision training used FP16 computation with gradient scaling. Computational-efficiency measurements used FP16 inference and batch size 1 (the batch size of 16 in **Table 2** refers exclusively to model training), GPU warm-up and CUDA synchronization.

The same hardware, numerical precision and software environment were used for the reimplemented baselines and the proposed framework. Classification stability with respect to the PV development partition was therefore assessed separately through the group-aware five-fold cross-validation reported in **Table 6**.

**Table 6:** Group-aware five-fold cross-validation results on the PV development cohort.

Fold	Held-out images	Accuracy (%)	Macro-F1 (%)
Fold 1	9,125	97.90	97.30
Fold 2	9,276	98.15	97.62
Fold 3	9,220	98.32	98.15
Fold 4	9,290	98.70	98.40
Fold 5	9,249	97.98	98.07
Mean ± sample SD	–	98.21 ± 0.32	97.91 ± 0.44

### 4.5 Hyperparameter configuration

**Table 2** distinguishes optimization settings from architectural and validation-selected inference settings. adaptively weighted AdamW training does not replace the internal AdamW update rule; it applies EMA normalization and smoothed adaptive weighting to the detection, segmentation and classification losses before the AdamW update. The EMA decay *ρ*, smoothing coefficient η and temperature τ were fixed before final evaluation.

The architectural query counts, classifier regularization settings and training budget were held constant across the controlled ablations. In **Table 3**, the fixed-weight and coordinated configurations with stage-specific gradients differed only in their task-weighting rule. In **Table 4**, optimizer-specific comparisons retained the same data partitions, model architecture, augmentations, validation schedule and maximum training budget. **Table 2** reports the final inference settings; their validation-only calibration and freezing procedure is described in Section 4.6.

### 4.6 Evaluation and comparison protocol

Model fitting was performed using the PV training partition, whereas early stopping, checkpoint selection, threshold calibration and post-processing selection were based exclusively on the validation partition. The leakage-controlled PV internal-test partition and independent field cohort remained excluded from model development. Final results were calculated separately for the two sources and were not pooled.

Inference-threshold and post-processing calibration was performed only after checkpoint selection. The detector-confidence threshold *τ*_det_ was selected on the PV detection-validation subset by maximizing class-agnostic leaf-localization F1 at an IoU matching threshold of 0.5. When candidate thresholds produced F1 values differing by no more than 1×10^−4^, the higher confidence threshold was selected to reduce false-positive proposals. This procedure yielded *τ*_det_ = 0.50. The maximum retained-RoI count was fixed at *R*_max_ = 300, equal to the number of RT-DETR queries, so every confidence-qualified final-layer prediction was retained.

The binary-mask threshold and lesion-mask refinement settings were selected using the PV segmentation-validation subset. Candidate configurations were compared primarily by lesion-positive Dice. When Dice values differed by no more than 1×10^−4^, the configuration with the lower verified-empty-mask FPR was selected. The retained configuration used *τ_m_* = 0.50, morphological opening followed by closing with a 3×3 elliptical structuring element and one iteration per operation, and *A*_min_ = 64 pixels.

Following calibration, all selected settings were frozen and applied without modification to the PV internal-test partition, independent field cohort, reimplemented baselines and controlled ablations. No PV internal-test or field images were used for threshold calibration or post-processing selection.

Classification was evaluated on the complete 38-category PV internal-test partition using accuracy, macro-precision, macro-recall and macro-F1. A source-matched analysis was additionally performed on PV test images belonging to the nine categories represented in the field cohort. The original 38-category output space was retained, and predictions assigned to any of the remaining categories were counted as errors. Macro metrics were calculated as unweighted averages of the corresponding category-specific values.

Detection was formulated as class-agnostic leaf localization. Precision, recall and F1 were calculated at the fixed detector-confidence threshold *τ*_det_ = 0.50, whereas AP@0.5 was obtained from ranked predictions at an IoU threshold of 0.5. Lesion segmentation was evaluated only on verified mask annotations. Dice, IoU, pixel precision and pixel recall were calculated on lesion-positive images by pooling pixel-level contingency counts within each source. Empty-mask images were evaluated separately using the proportion producing at least one false lesion region after fixed thresholding and post-processing.

Segmentation evaluation used RT-DETR-predicted leaf RoIs. Predicted masks were projected to the original image coordinates, and localization failures were retained as empty lesion predictions. Reimplemented baselines used the same source-separated manifests, annotations, validation-only selection procedure and evaluation code. Controlled ablations used matched configurations that differed only in the component or training strategy under investigation.

Group-aware five-fold cross-validation was conducted only within the PV development cohort to assess classification stability; the fixed internal-test and field cohorts remained excluded.

### 4.7 Computational complexity analysis

The computational requirements of the proposed framework arise from RT-DETR localization, Mask2Former lesion segmentation and EfficientNet-B4 classification with channel-attention refinement. Theoretical complexity expressions were used to describe how the principal attention and convolutional operations scale with feature dimensions. Actual parameters, GFLOPs, latency and throughput were measured from the implemented framework and are reported separately in Section 5.8.

Let *n* denote the number of feature tokens and (d) denote the feature dimension. The dominant token-to-token interaction cost of self-attention is approximated using Equation 67,

(67) $$O_{attn}=\;O\left(n^{2}d\right)$$

RT-DETR reduces the need to apply global attention directly to every high-resolution feature map through its hybrid encoder and query-based decoder. It also avoids anchor enumeration and non-maximum suppression. Mask2Former applies masked attention so that each segmentation query aggregates features primarily from spatial locations associated with its current lesion-mask prediction. Masked attention improves spatial selectivity, although its measured computational benefit depends on whether the implementation exploits sparse operations.

EfficientNet-B4 performs feature extraction using standard, depthwise and pointwise convolutions. For a grouped convolution, the computational complexity is approximated using Equation 68,

(68) $$O_{conv}=O\left(\frac{hwk^{2}C_{in}C_{out}}{g}\right)$$

where *h* and *w* are the output feature-map dimensions, *k* is the kernel size, *C_in_* and *C_out_* are the input and output channels, and *g* is the number of convolution groups. For standard convolution, *g* = 1 for depthwise convolution; *g* = *C_in_*. Channel-attention refinement and the final classification head introduce additional operations that were included in the measured classifier cost.

The complete-framework parameter count is obtained from the unique stored weights of its three principal modules using Equation 69,

(69) $$P_{total}=\;P_{det}+P_{seg}+P_{cls}$$

where *P_det_*, *P_seg_* and *P_cls_* denote the parameter counts of the RT-DETR, Mask2Former and EfficientNet-B4 classification modules, respectively. Parameters are counted once and are not multiplied by the number of detected RoIs because the same segmentation and classification weights are reused for every processed leaf region.

## 5 Results and discussion

This section reports disease-classification, class-agnostic leaf-localization, lesion-segmentation, ablation, computational-efficiency, qualitative and statistical results. Classification, localization and segmentation operate on different prediction units and annotation subsets; their metrics were therefore calculated and interpreted independently rather than combined into a single performance measure. PV and independently acquired field data were also evaluated separately to distinguish controlled-domain performance from field-domain generalization.

### 5.1 Classification metrics

Disease classification was evaluated using overall accuracy together with macro-precision, macro-recall, and macro-F1. For each disease category *c*, precision, recall, and F1-score were computed using Equation 70,

(70) $$\begin{array}{c} Precision_{c}=\;\frac{TP_{c}}{TP_{c}+FP_{c}}\\ Recall_{c}=\;\frac{TP_{c}}{TP_{c}+FN_{c}}\\ F1_{c}=\;\frac{2\times Precision_{c}\times Recall_{c}}{Precision_{c}+Recall_{c}} \end{array}$$

where *TP_c_*, *FP_c_*, and *FN_c_* denote the true-positive, false-positive, and false-negative predictions for category *c*, respectively. For *C* evaluated categories, the macro-averaged measures were obtained from Equation 71,

(71) $$\begin{array}{c} Macro-Precision=\;\frac{Precision_{1}+Precision_{2}+\cdots \cdots \cdots \cdots +Precision_{C}}{C}\\ Macro-Recall=\;\frac{Recall_{1}+Recall_{2}+\cdots \cdots \cdots \cdots +Recall_{C}}{C}\\ Macro-F1=\;\frac{F1_{1}+F1_{2}+\cdots \cdots \cdots \cdots +F1_{C}}{C} \end{array}$$

while overall classification accuracy was calculated using Equation 72,

(72) $$Accuracy=\;\frac{Number\;of\;correctly\;classified\;images}{Total\;number\;of\;test\;images}\times 100$$

Macro-F1 was therefore calculated as the arithmetic mean of the class-wise F1-scores and not as the harmonic mean of macro-precision and macro-recall. This formulation gives equal contribution to each disease category and reduces the influence of unequal category support on the reported summary measure.

### 5.2 Leaf-localization metrics

RT-DETR was evaluated as a class-agnostic leaf localizer. A predicted bounding box was treated as a true-positive detection when it satisfied the prescribed intersection-over-union matching criterion with a ground-truth leaf instance; unmatched predictions and unmatched ground-truth instances were counted as false positives and false negatives, respectively. Detection precision, recall, and F1-score were calculated from Equation 73,

(73) $$\begin{array}{c} Precision_{det}=\;\frac{TP_{det}}{TP_{det}+FP_{det}}\\ Recall_{det}=\;\frac{TP_{det}}{TP_{det}+FN_{det}}\\ F1_{det}=\;\frac{2\times Precision_{det}\times Recall_{det}}{Precision_{det}+Recall_{det}} \end{array}$$

where *TP_det_* denotes correctly localized leaves, *FP_det_* and *FN_det_* represent incorrect leaf detections and missed leaves respectively. Localization quality over the confidence-ranked detections was additionally summarized using average precision at an IoU threshold of 0.5 using Equation 74,

(74) AP@0.5=Area under the Precision−Recall curve at IoU≥0.5

### 5.3 Lesion-segmentation metrics

Lesion segmentation was evaluated after projecting the predicted masks from the RT-DETR leaf RoIs back to the original image coordinate system. For lesion-positive images, pixel-level true-positive, false-positive, and false-negative counts were pooled within each evaluation source. Let *TP_pixel_*, *FP_pixel_*, and *FN_pixel_* denote these aggregated pixel counts. Pixel precision and recall were defined using Equation 75,

(75) $$\begin{array}{c} Pixel\;Precision=\frac{TP_{pixel}}{TP_{pixel}+FP_{pixel}}\\ Pixel\;Recall=\frac{TP_{pixel}}{TP_{pixel}+FN_{pixel}} \end{array}$$

where *TP_pixel_* denotes lesion pixels correctly predicted as lesion, *FP_pixel_*, and *FN_pixel_* represents non-lesion pixels incorrectly predicted as lesion and actual lesion pixels missed by the model respectively. The Dice coefficient and intersection-over-union were calculated from Equation 76,

(76) $$\begin{array}{c} Dice=\;\frac{2TP_{pixel}}{2TP_{pixel}+FP_{pixel}+FN_{pixel}}\\ IoU=\;\frac{TP_{pixel}}{TP_{pixel}+FP_{pixel}+FN_{pixel}} \end{array}$$

The empty-mask false-positive rate is defined from Equation 77,

(77) $$Empty-mask\;FPR=\frac{Number\;of\;empty-mask\;images\;predicted\;with\;a\;lesion}{Total\;number\;of\;empty-mask\;images}\times 100$$

This measure quantifies erroneous lesion activation on lesion-free leaves independently of lesion-positive overlap performance.

### 5.4 Computational-efficiency metrics

Computational efficiency was characterized using total and trainable parameter counts, GFLOPs, mean inference latency, and frames per second (FPS). For *N* sequentially processed images with inference times *t_i_*, mean latency and throughput were calculated using Equation 78,

(78) $$\begin{array}{c} Mean\;Latency=\frac{t_{1}+t_{2}+\cdots \cdots \cdots \cdots +t_{N}}{N}\\ FPS=\;\frac{1000}{Mean\;Latency\left(ms\right)} \end{array}$$

### 5.5 Quantitative performance comparison

**Tables 7A**-**9B** present the source-separated classification, leaf-localization and lesion-segmentation results obtained under the evaluation protocol defined in Section 4.6.

**Table 7A:** Source-separated classification performance of the proposed framework.

Evaluation cohort	Categories	Test images	Accuracy (%)	Macro-precision (%)	Macro-recall (%)	Macro-F1 (%)
Complete PV internal test	38	8,146	98.34	97.60	98.39	97.97
Category-matched PV internal-test subset	9	1,828	97.43	96.89	96.72	96.77
Independent field cohort	9	4,200	84.93	81.49	80.94	80.80

**Table 7B:** Classification comparison under the common experimental protocol.

Method	PV-38 accuracy (%)	PV-38 macro-F1 (%)	Matched PV-9 macro-F1 (%)	Field-9 accuracy (%)	Field-9 macro-F1 (%)
ResNet50 (He et al., 2016)	92.11	91.58	88.07	75.07	72.18
EfficientNet-B4 (Tan and Le, 2019)	94.05	92.14	90.46	79.21	71.03
RT-DETR RoI + EfficientNet-B4 without lesion-mask guidance (Zhao et al., 2024; Tan and Le, 2019)	95.26	92.97	89.63	79.60	73.97
Adapted PDLC-ViT shared-encoder segmentation–classification baseline (Hemalatha and Jayachandran, 2024)	96.12	93.85	91.79	78.95	74.84
Proposed RT-DETR–Mask2Former framework	98.34	97.97	96.77	84.93	80.80

**Table 8A:** Source-separated performance of the proposed leaf-localization module.

Evaluation cohort	Annotated images	Bounding-box instances	Precision (%)	Recall (%)	F1-score (%)	AP@0.5 (%)
PV internal-test subset	1,614	2,018	94.37	92.84	93.60	95.63
Independent field cohort	4,200	5,250	85.97	83.56	84.75	89.28

**Table 8B:** Detection comparison under the common experimental protocol.

Reimplemented detector	PV F1-score (%)	PV AP@0.5 (%)	Field F1-score (%)	Field AP@0.5 (%)	FPS
Faster R-CNN - ResNet50 - FPN (Ren et al., 2017; He et al., 2016; Lin et al., 2017)	85.73	87.08	71.50	76.88	17
YOLOv8m (Jocher et al., 2023)	87.48	88.96	75.43	81.92	35
RT-DETR-R50 (Zhao et al., 2024)	90.12	90.93	79.90	85.94	25
YOLOv10-M (Wang et al., 2024)	89.76	90.08	78.89	84.93	40
RT-DETRv3-R50 (Wang et al., 2025)	91.27	91.86	80.91	86.30	24
Proposed RT-DETR localization variant	93.60	95.63	84.75	89.28	36

**Table 9A:** Source-separated performance of the proposed lesion-segmentation module.

Evaluation cohort	PV annotated internal-test subset	Independent annotated field cohort
Annotated images	858	4,200
Lesion-positive images	763	3,500
Empty-mask images	95	700
Ground-truth mask instances	1,073	5,250
Dice (%)	92.47	77.90
IoU (%)	86.00	63.80
Pixel precision (%)	92.98	78.92
Pixel recall (%)	91.97	76.90
Empty-mask FPR (%)	3.16	4.86

**Table 9B:** Comparison of lesion-segmentation configurations under the common source-separated evaluation protocol.

Segmentation configuration	PV Dice (%)	PV IoU (%)	PV empty-mask FPR (%)	Field Dice (%)	Field IoU (%)	Field empty-mask FPR (%)
U-Net (Ronneberger et al., 2015)	82.88	70.77	7.37	58.69	41.53	20.34
DeepLabV3+-ResNet50 (Chen et al., 2018; He et al., 2016)	86.91	76.85	4.21	65.76	48.99	13.28
PSPNet–ResNet50 (Zhao et al., 2017; He et al., 2016)	85.90	75.28	5.26	63.74	46.78	15.20
Mask2Former–Swin-B (Cheng et al., 2022; Liu et al., 2021)	89.93	81.70	4.21	71.81	56.02	10.92
Proposed RT-DETR- Mask2Former framework	92.47	86.00	3.16	77.90	63.80	4.86

#### 5.5.1 Classification performance

The PV results in **Table 7A** show that the lesion-guided representation preserved high classification performance on the complete PV internal-test partition and its category-matched subset. The high macro-F1 indicates that performance was distributed across categories rather than being determined only by categories with large image supports. The field results demonstrate reduced generalization under independent acquisition. This behavior is compatible with changes in background composition, illumination, leaf pose, lesion scale and symptom presentation, although the effect of each factor cannot be isolated without condition-specific annotations.

**Table 7B** compares classifiers trained and evaluated under a common experimental protocol. ResNet50 and EfficientNet-B4 operate on complete images and must therefore extract disease-discriminative features while suppressing responses arising from healthy tissue and background variability. Restricting EfficientNet-B4 to an RT-DETR-predicted leaf region reduces gross background interference, but the localized region can still contain substantial healthy tissue and non-diagnostic visual structures. Consequently, localization improves spatial focus but does not independently eliminate intra-leaf feature ambiguity. The localization-only configuration therefore exhibits metric-specific behavior: accurate leaf localization can reduce background-related errors without necessarily improving the separability of diseases with visually similar lesion patterns.

The proposed configuration additionally uses the predicted lesion mask to construct a disease-focused representation before EfficientNet-B4 classification. The mask provides an explicit spatial prior that concentrates feature extraction on symptom-bearing tissue, while channel attention emphasizes feature channels associated with lesion texture, color and structural irregularity. This combination reduces reliance on background correlations and non-lesion leaf appearance. The stronger macro-F1 observed for the proposed model is therefore consistent with more balanced category-level discrimination rather than improvement confined to dominant categories.

The adapted PDLC-ViT baseline learns a shared encoder representation for segmentation and classification tasks having different spatial objectives. By contrast, the proposed pipeline explicitly separates leaf localization, lesion delineation, and lesion-guided classification. During forward processing, RT-DETR detections define the leaf RoIs, Mask2Former masks are applied to the corresponding RoIs, and the resulting lesion-conditioned RoIs are passed downstream to EfficientNet-B4. Thus, stage outputs are passed downstream as data inputs, but gradients do not propagate across the module boundaries. Under the common experimental protocol, the observed results are consistent with the benefit of an explicit lesion-conditioned representation over reliance solely on implicit feature sharing. This interpretation is supported primarily by the matched mask-guidance ablation rather than by comparisons with independently structured baselines.

**Figure 6** presents the nine ground-truth categories shared by the matched PlantVillage and independent field cohorts. Predictions assigned to any of the remaining 29 categories in the retained 38-category output space are combined in the “other 29” column and counted as errors. The field confusion matrix shows concentrated errors between visually related disease categories, consistent with the observed reductions in macro-recall and macro-F1. In particular, confusion involving Septoria leaf spot and Early blight is compatible with partial similarity in necrotic spot appearance when lesion scale, contrast or disease severity varies.

**Figure 6:**
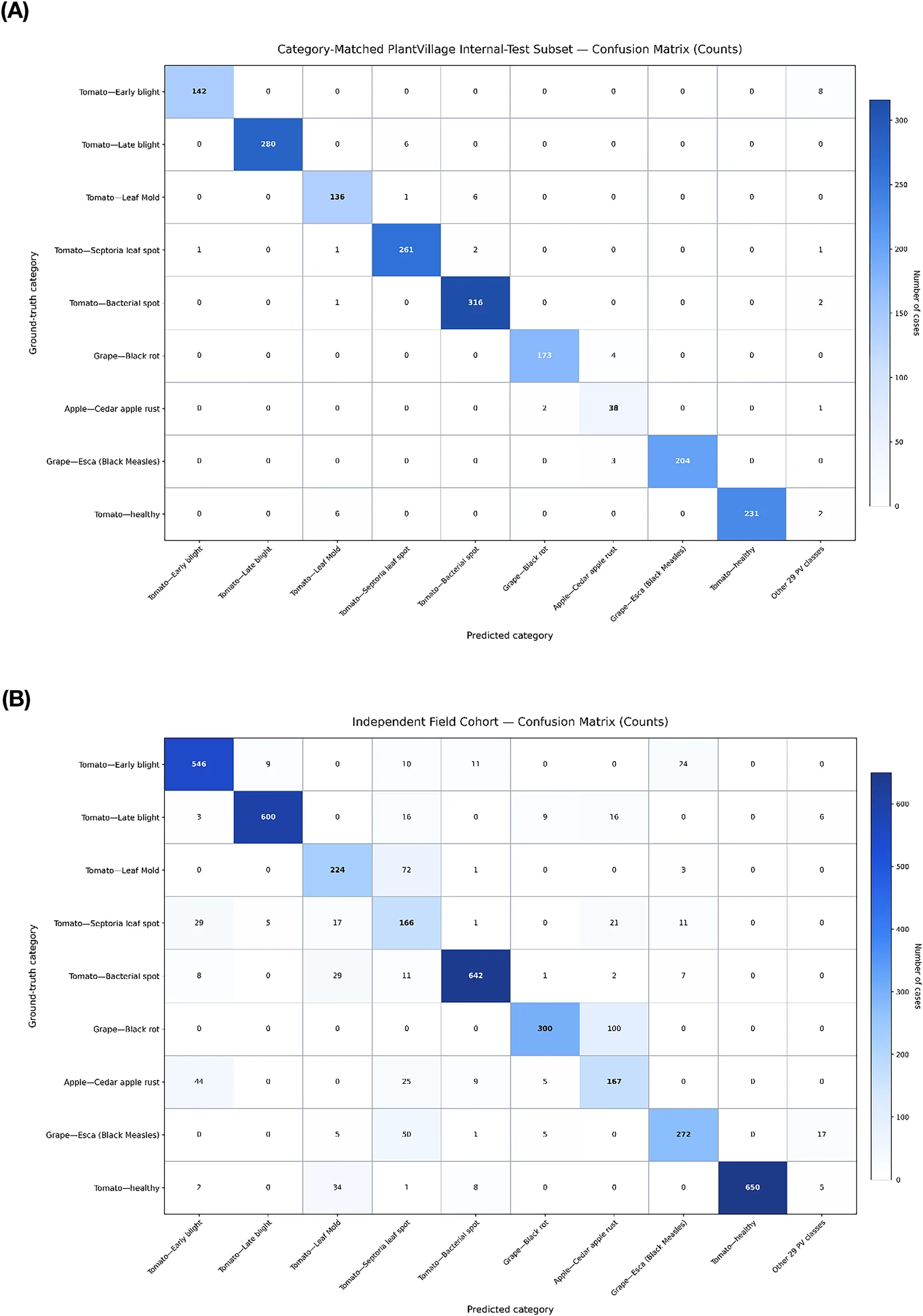
Confusion matrices for **(A)** Category-matched PV internal-test subset and **(B)** Independent field cohort.

#### 5.5.2 Detection performance

**Table 8A** shows that RT-DETR acts as a class-agnostic leaf localizer to identify leaf regions and confidence scores, whereas disease-category prediction is performed downstream by EfficientNet-B4. The simultaneous deterioration of precision and recall in the field cohort indicates both spurious leaf proposals and missed leaf instances. Such an error pattern is compatible with cluttered vegetation, overlapping leaves, weak boundaries and variations in object scale.

The comparison in **Table 8B** reflects the different localization mechanisms of the detectors. Faster R-CNN uses a two-stage proposal and refinement process, which provides explicit region refinement but introduces sequential computation and dependence on proposal quality. YOLOv8m and YOLOv10-M use dense one-stage prediction and provide high throughput, but their local prediction structure can remain sensitive to cluttered backgrounds and overlapping objects. RT-DETR instead combines multi-scale feature encoding with query-based set prediction and one-to-one matching, allowing leaf candidates to be selected using broader image context while reducing redundant detections.

The proposed localization variant produced the strongest point estimates on both sources while maintaining real-time detector-level throughput. This pattern is consistent with the ability of the RT-DETR representation to integrate local boundary evidence with global contextual information. The measured detector throughput should also be distinguished from the lower throughput of the complete segmentation-classification pipeline.

#### 5.5.3 Lesion segmentation performance

**Table 9A** reports lesion segmentation on lesion-positive and lesion-free images separately. Dice, IoU, pixel precision and pixel recall were calculated from pixel-level counts pooled within each source and are therefore source-level micro-averaged estimates. Empty-mask images were excluded from the overlap metrics and evaluated through empty-mask FPR, preventing correctly predicted healthy images from receiving artificial perfect-overlap scores.

Segmentation was performed on RT-DETR-predicted leaf regions rather than ground-truth regions. Failed localization therefore resulted in a retained empty lesion prediction, and all masks were projected back to the original image coordinates before evaluation. The reported segmentation results consequently measure the deployed localization-segmentation pathway and include errors introduced by inaccurate or missing leaf regions.

The lower field overlap metrics reflect a combination of missed lesion pixels, spurious foreground responses and upstream localization error. Pixel recall was slightly lower than pixel precision, indicating that lesion omission was marginally more prominent than foreground expansion. The higher empty-mask FPR shows that field-domain texture or background structures more frequently activated the lesion predictor on healthy images. Because the inference configuration was calibrated exclusively on PV validation data under the procedure in Section 4.6, the field results do not reflect field-specific threshold or post-processing optimization.

The comparisons in **Table 9B** represent complete lesion-segmentation configurations that differ in both segmentation architecture and spatial input. U-Net, DeepLabV3+–ResNet50, PSPNet–ResNet50 and Mask2Former–Swin-B received the complete image resized to 640×640 pixels. In contrast, the proposed configuration received an RT-DETR-predicted leaf RoI expanded by δ=0.10 and resized to the same resolution. After inference, the masks generated by the complete-image configurations were inverse-resized, whereas the RoI-level mask produced by the proposed configuration was projected back to its corresponding location in the original image. All metrics were subsequently calculated on the original-image pixel grid using the same source-specific evaluation manifests. A missed or rejected leaf detection in the proposed pipeline was retained as an empty lesion prediction. Dice and IoU were calculated on lesion-positive images, while the empty-mask false-positive rate was calculated on lesion-free images.

Architecturally, U-Net reconstructs lesion masks through convolutional encoder–decoder features and skip connections. DeepLabV3+ and PSPNet enlarge contextual coverage through atrous convolution and pyramid pooling, respectively, whereas Mask2Former–Swin-B employs query-based mask prediction and masked attention to represent spatially irregular lesions. The proposed configuration combines query-based segmentation with detector-conditioned RoI processing. Restricting inference to the localized leaf region reduces the area in which lesion candidates are evaluated and limits exposure to unrelated background structures. The higher overlap scores and lower empty-mask false-positive rate reported for the proposed configuration are therefore consistent with the combined effects of spatially focused input, query-based lesion representation and fixed mask refinement.

Although the predicted lesion masks permit computation of lesion-to-leaf area ratios, the analysis in **Table 9B** evaluates only spatial segmentation performance. The resulting area measurements were not interpreted as validated disease-severity estimates because the present dataset did not contain prespecified severity categories, expert ordinal severity scores or longitudinal disease-progression measurements. Consequently, no agreement analysis between mask-derived lesion area and plant-pathologist severity assessment was performed.

### 5.6 Ablation analysis

A controlled ablation analysis was conducted to determine whether the observed classification improvement arose from leaf localization, lesion-mask guidance or their combined use. All ablation configurations used the same data partitions, classifier output space, initialization, optimization schedule, augmentation policy and checkpoint-selection rule.

#### 5.6.1 Downstream effects of localization and lesion guidance

**Table 10** shows that leaf localization alone has an endpoint-dependent effect. RoI extraction suppresses external background and normalizes leaf scale, but predicted boxes can exclude peripheral symptoms or remove contextual information that contributes to classification. Detection jitter can also alter the crop presented to EfficientNet-B4. These competing effects explain why cropping alone does not uniformly improve every cohort and metric.

**Table 10:** Downstream classification ablation of localization and lesion-mask guidance.

Configuration	Classification input	Predicted leaf RoI	Predicted lesion mask	PV-38 accuracy (%)	PV-38 macro-F1 (%)	Matched PV-9 macro-F1 (%)	Field-9 accuracy (%)	Field-9 macro-F1 (%)
EfficientNet-B4 whole-image baseline	Complete image	No	No	94.05	92.14	90.46	79.21	71.03
RT-DETR RoI + EfficientNet-B4	Localized leaf region	Yes	No	95.26	92.97	89.63	79.60	73.97
Proposed RT-DETR+Mask2Former+EfficientNet-B4	Lesion-guided localized leaf region	Yes	Yes	98.34	97.97	96.77	84.93	80.80

Adding predicted lesion guidance produced consistently stronger classification point estimates. Unlike a bounding box, which identifies the complete leaf region, the lesion mask distinguishes symptom-bearing pixels from visually non-diagnostic tissue. The resulting representation directs EfficientNet-B4 toward lesion color, texture, shape and distribution while limiting dependence on healthy tissue and environmental background. The consistent pattern across the classification endpoints supports the diagnostic value of this lesion-conditioned representation.

#### 5.6.2 Localization and segmentation error-propagation controls

**Table 1** uses ground-truth leaf regions and lesion masks as diagnostic controls. Replacing the ground-truth leaf region with the RT-DETR prediction isolates the downstream consequence of localization error under the mask-free configuration. Holding the predicted leaf region fixed and replacing the ground-truth lesion mask with the Mask2Former prediction isolates the effect associated with segmentation error.

The larger oracle-to-predicted mask separation in the field cohort shows that segmentation imperfections had a greater downstream effect under source variation. Nevertheless, the predicted-mask pipeline remained stronger than the corresponding predicted-RoI configuration without lesion guidance, indicating that imperfect lesion masks still retained diagnostically useful spatial information.

#### 5.6.3 Multi-task training-strategy ablation

Sequential module-wise training fixed each upstream checkpoint before optimizing the subsequent module. In the coordinated configurations, the three modules were updated within the common schedule defined in Section 3.8, while retaining disjoint module-specific gradient paths. The two coordinated configurations differed only in their task-loss weighting rule: fixed equal weighting assigned $w_{k}=1$ to each active EMA-normalized loss, whereas adaptive weighting varied the coefficients according to recent within-task loss behavior.

Relative to fixed equal weighting, adaptive weighting increased the PV leaf-localization AP@0.5, lesion Dice and matched-PV macro-F1 point estimates by 0.68%, 1.55% and 0.80%, respectively. The corresponding field differences were 1.39%, 3.12% and 1.99%. These comparisons evaluate the effect of adaptive scaling of separately supervised objectives and should not be interpreted as evidence of cross-module gradient propagation or shared-backbone gradient-conflict resolution.

### 5.7 Effect of EMA-normalized adaptive task-loss weighting

**Table 4** separates the effects of the base optimizer and adaptive task weighting under the common training protocol. With fixed equal task weights, SGD applies the shared learning-rate schedule without parameter-wise moment estimation. Adam incorporates first- and second-moment estimates to accommodate heterogeneous and noisy gradients, whereas AdamW decouples weight decay from the gradient-based parameter update and provides more direct control over regularization.

AdamW with EMA-normalized adaptive task-loss weighting retains AdamW as the base optimizer while applying EMA normalization and smoothed adaptive weighting to the applicable detection, segmentation and classification losses. In the fixed-weight AdamW configuration, the same normalized task losses and training settings were retained, but each active task was assigned $\omega_{k}=1$. The comparison between fixed-weight AdamW and AdamW with EMA-normalized adaptive task-loss weighting therefore evaluates the contribution of the adaptive weighting rule without changing the optimizer, data partitions, augmentation schedule, learning-rate schedule or checkpoint-selection criterion.

With the procedure defined in Section 3.8, EMA-normalized adaptive weighting modifies only the scalar coefficients applied to the task-specific losses before the standard AdamW update. **Table 4** therefore isolates the effect of the base optimizer and task-loss-weighting rule without implying cross-module backpropagation.

The convergence epoch denotes the epoch attaining the minimum mean EMA-normalized PV validation loss under the checkpoint-selection rule defined in Section 4.3. Validation was performed once per epoch, and ties within 1×10^−4^ were resolved by retaining the earlier checkpoint. Because all configurations used identical numbers of global optimizer updates per epoch, validation frequency, early-stopping patience and maximum training budget, an earlier selected checkpoint is consistent with more rapid attainment of the validation optimum.

### 5.8 Computational efficiency analysis

Computational efficiency was evaluated at component and complete-pipeline levels. All task-matched configurations were profiled on the same NVIDIA RTX 4090 platform using 640 × 640-pixel inputs, FP16 precision and inference batch size 1. Measurements were obtained after GPU warm-up and CUDA synchronization using the same evaluation manifest, RoI-retention rule, GFLOP-counting convention and timing boundaries. The batch size of 16 reported in **Table 2** applies to model training and is independent of the batch-size-1 protocol used to measure latency and FPS.

End-to-end latency included preprocessing, host-to-device transfer, RT-DETR localization, bounding-box post-processing, RoI extraction, Mask2Former segmentation, mask refinement and projection, EfficientNet-B4 classification, final post-processing and synchronization. These operations were included in their corresponding integrated component times. Consequently, the detector-level FPS reported in **Table 8B** and complete-pipeline FPS reported in **Table 11B** represent different computational scopes.

Total parameters represent all unique model weights stored in the assembled framework, whereas trainable parameters include only the weights updated during fine-tuning. Based on the component values reported in **Table 11A**, the total parameter count is 106M. The corresponding trainable parameter count is 87M. Parameter count remains unchanged when multiple leaf RoIs are processed because the same Mask2Former and EfficientNet-B4 weights are reused for each RoI.

**Table 11A:** Component-wise computational requirements of the proposed framework.

Component	Invocation basis	Total parameters (M)	Trainable parameters (M)	GFLOPs	Mean latency per invocation (ms)
RT-DETR localization module	Per input image	42	36	136	≈28
Mask2Former segmentation module	Per processed leaf RoI	44	35	110	≈20
EfficientNet-B4 + Channel attention + Classification head		20	16	9	≈8
Complete framework	One image and one processed leaf RoI	106	87	255	≈56

**Table 11B:** End-to-end efficiency comparison under the common timing protocol.

End-to-end configuration	Total parameters (M)	Mean GFLOPs/image	Mean end-to-end latency (ms)	FPS
Faster R-CNN–ResNet50-FPN + U-Net + EfficientNet-B4	116	255	90	≈11
YOLOv8m + DeepLabV3+ResNet50 + EfficientNet-B4	109	289	44	≈23
RT-DETR–ResNet50 + Mask2Former–Swin-B + EfficientNet-B4	178	407	72	≈14
Proposed RT-DETR + Mask2Former + EfficientNet-B4 with channel attention refinement	106	285	63	≈16

**Table 11A** reports component-level costs for one invocation. For an image containing one retained leaf RoI, the localization, segmentation and classification modules are each executed once, giving 255 GFLOPs and an integrated latency of approximately 56 ms. Across the common timing manifest, the proposed detector retained an average of 1.25 RoIs per image. Because RT-DETR is invoked once per image, whereas Mask2Former and EfficientNet-B4 are each invoked once per retained RoI, the corresponding dataset-level costs are approximately 285 GFLOPs and 63 ms per image. Model parameters are not duplicated across RoIs because the same trained weights are reused.

For synchronized batch-size-1 execution, throughput was derived from the total elapsed time as 16 FPS. The detector-only latency of approximately 28 ms corresponds to 36 FPS. The 36-FPS result in **Table 8B** therefore characterizes only RT-DETR localization and bounding-box post-processing; it is not the throughput of the complete localization–segmentation–classification pipeline.

**Table 11B** compares only configurations that perform all three tasks. The YOLOv8m–DeepLabV3+ configuration provides the highest throughput, whereas the proposed configuration occupies an intermediate operating point: it is faster than the Faster R-CNN–U-Net and RT-DETR–Mask2Former–Swin-B configurations but slower than the YOLOv8m-based pipeline. The proposed configuration nevertheless has the lowest parameter count among the evaluated end-to-end systems and requires approximately 30% fewer GFLOPs per image than the Swin-B configuration. GFLOPs represent arithmetic operation count, whereas latency is additionally affected by memory transfer, kernel implementation, intermediate-feature dimensions, hardware utilization and synchronization overhead. This explains why the Faster R-CNN-based configuration reports fewer GFLOPs but has higher latency than the proposed framework. Overall, the results characterize the proposed framework as a task-matched trade-off among predictive performance, lesion-level diagnostic output and computational cost.

### 5.9 Qualitative analysis

Qualitative inspection was performed as a descriptive complement to the source-separated quantitative evaluation. Representative test examples were processed using the deployable inference configuration, in which the downstream modules received RT-DETR-predicted leaf RoIs and Mask2Former-predicted lesion masks. Ground-truth RoIs or masks were not substituted during this analysis.

**Figure 7** presents the sequential outputs for three representative disease cases: tomato early blight, tomato late blight and grape black rot. For each example, the figure shows the original image, RT-DETR leaf localization, the Mask2Former lesion-mask overlay, the lesion-isolated representation supplied to the classifier and the final EfficientNet-B4 top-1 prediction.

**Figure 7:**
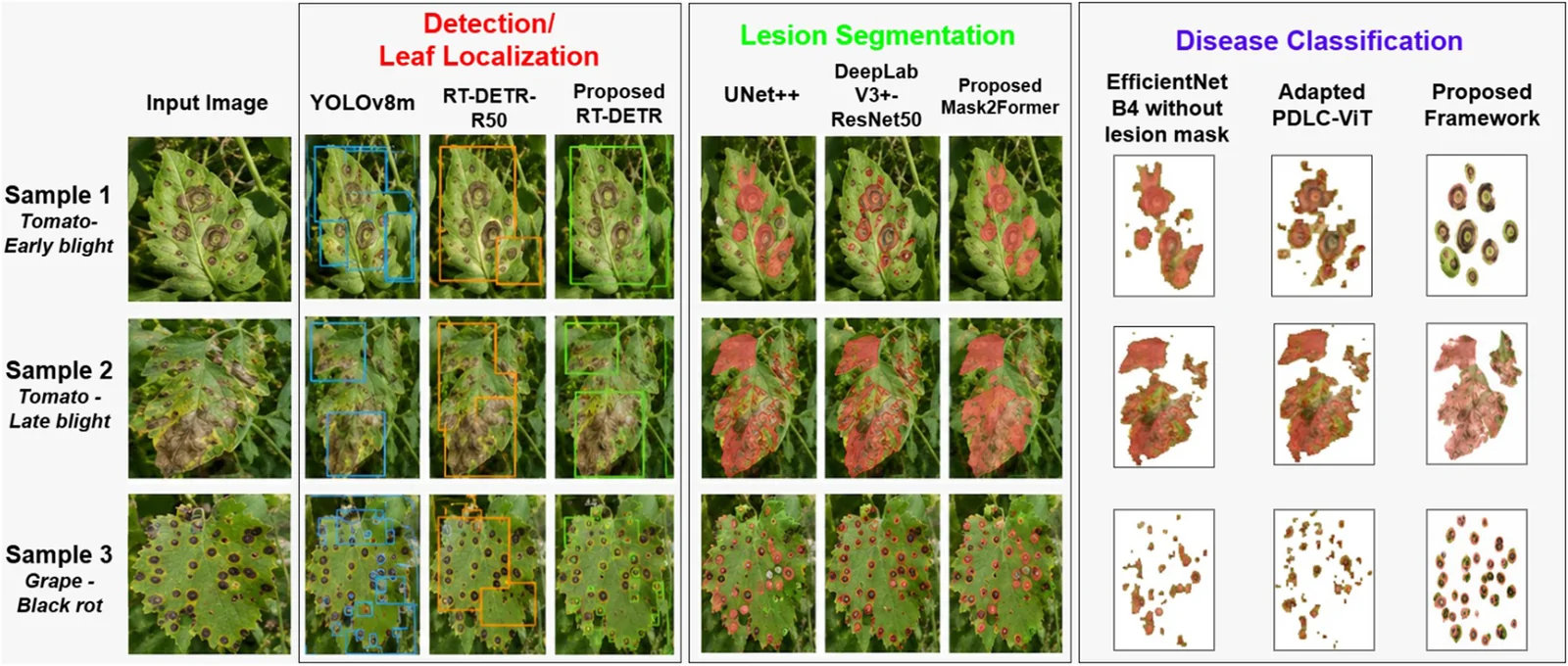
Qualitative comparison of the proposed RT-DETR–Mask2Former framework showing leaf localization, lesion segmentation, disease-region isolation, and disease classification from isolated lesion regions on representative test samples.

The localization results show that the predicted RoIs retained the principal disease-bearing leaf regions despite background vegetation, irregular leaf orientation and differences in image scale. The segmentation overlays further demonstrate the response of Mask2Former to distinct lesion morphologies. The early-blight and black-rot examples contain multiple spatially separated, approximately circular lesions, whereas the late-blight example presents a larger and more diffuse necrotic region. In each case, the predicted mask preserved the visible distribution of the symptomatic tissue while suppressing much of the surrounding healthy leaf area and image background.

The resulting lesion-isolated representations provide an interpretable intermediate output showing the image evidence supplied to the disease classifier. The corresponding top-1 predictions were tomato early blight, tomato late blight and grape black rot for the three illustrated cases. These examples demonstrate the operational relationship among localization, lesion segmentation and lesion-conditioned classification. However, they should be interpreted as descriptive examples rather than estimates of population-level performance. The quantitative contribution of localization and lesion guidance is evaluated through the controlled ablations, whereas predictive performance and domain generalization are assessed using the source-separated results reported in **Tables 7A**, **8A**, **9A**.

### 5.10 Statistical validation analysis

**Table 6** evaluates classification stability across group-aware five-fold cross-validation within the PV development cohort. Retaining duplicate and leaf groups within a single fold limits information leakage from related images. The close fold-wise results indicate that controlled-domain classification was not strongly dependent on one particular development partition. Macro-F1 varies more than accuracy because it gives equal importance to every category and is therefore more sensitive to category composition and low-support class errors. The cross-validation results support the stability of PV classification. The cross-validation results support the stability of PV classification only. They do not establish the stability of the detection and segmentation modules, which would require corresponding task-specific cross-validation or repeated external evaluation.

## 6 Conclusion and future work

In this study, an RT-DETR-Mask2Former framework was proposed for class-agnostic leaf localization, lesion segmentation and lesion-guided disease classification in agricultural images. The separately supervised task objectives were coordinated through EMA normalization and smoothed adaptive weighting under the disjoint module-specific gradient paths. Model development was restricted to leakage-controlled, group-aware PV training and validation partitions, while the independent field cohort remained excluded from model and parameter selection.

On the complete 38-category PV internal test, the framework achieved 98.34% accuracy and 97.97% macro-F1. On the nine-category source-matched subset, accuracy and macro-F1 were 97.43% and 96.77%, compared with 84.93% and 80.80% in the field cohort. Leaf-localization AP@0.5 decreased from 95.63% to 89.28%, while lesion-positive Dice/IoU decreased from 92.47%/86.00% to 77.90%/63.80%. Controlled ablation showed that predicted lesion guidance increased field macro-F1 from 73.97% to 80.80% relative to predicted-RoI classification without mask guidance. At a mean of 1.25 processed RoIs per image, the complete pipeline required approximately 285 GFLOPs and 63 ms per image, corresponding to 16 FPS under batch-size-1 FP16 inference. Generalizability remains limited by the single field cohort and nine shared categories. The observed field degradation is compatible with missed leaves, inaccurate RoIs, small or low-contrast lesions, overlap and background clutter.

Future work should prioritize multi-scale and boundary-aware refinement for small or fragmented lesions, multi-location and multi-season external validation, and domain-generalization or source-free adaptation evaluated on unseen crops and sites. Distillation, pruning, quantization and hardware-specific acceleration should be evaluated for edge, mobile and UAV deployment under explicit memory, energy and latency constraints. Future work will validate lesion-to-leaf area ratios against blinded assessments by plant pathologists and repeated measurements of disease progression before their use in agronomic decision-making.

## Data Availability

The publicly available PlantVillage dataset can be accessed from its original source (https://www.tensorflow.org/datasets/catalog/plant_village). The additional field-acquired images, boundingbox annotations, lesion-mask annotations, and derived datasets generated during this study are available from the corresponding author upon reasonable request.
